# Structural and mechanistic insights into the DNA glycosylase AAG-mediated base excision in nucleosome

**DOI:** 10.1038/s41421-023-00560-0

**Published:** 2023-06-20

**Authors:** Lvqin Zheng, Bin Tsai, Ning Gao

**Affiliations:** 1 State Key Laboratory of Membrane Biology, Peking-Tsinghua Center for Life Sciences, School of Life Sciences, Peking University, Beijing, China; 2 Academy for Advanced Interdisciplinary Studies, Peking University, Beijing, China

**Keywords:** Electron microscopy, DNA damage and repair

## Abstract

The engagement of a DNA glycosylase with a damaged DNA base marks the initiation of base excision repair. Nucleosome-based packaging of eukaryotic genome obstructs DNA accessibility, and how DNA glycosylases locate the substrate site on nucleosomes is currently unclear. Here, we report cryo-electron microscopy structures of nucleosomes bearing a deoxyinosine (DI) in various geometric positions and structures of them in complex with the DNA glycosylase AAG. The apo nucleosome structures show that the presence of a DI alone perturbs nucleosomal DNA globally, leading to a general weakening of the interface between DNA and the histone core and greater flexibility for the exit/entry of the nucleosomal DNA. AAG makes use of this nucleosomal plasticity and imposes further local deformation of the DNA through formation of the stable enzyme–substrate complex. Mechanistically, local distortion augmentation, translation/rotational register shift and partial opening of the nucleosome are employed by AAG to cope with substrate sites in fully exposed, occluded and completely buried positions, respectively. Our findings reveal the molecular basis for the DI-induced modification on the structural dynamics of the nucleosome and elucidate how the DNA glycosylase AAG accesses damaged sites on the nucleosome with different solution accessibility.

## Introduction

The eukaryotic base excision repair (BER) machinery locates and repairs DNA base damage in chromatin^1,2^. Genomic DNA contains substantial amounts of DNA base damages due to various exogenous damaging agents and spontaneous decomposition reactions — such as deamination of deoxycytidine (DC, cytosine) to deoxyuridine (DU, uracil) and deamination of deoxyadenosine (DA, adenine) to deoxyinosine (DI, hypoxanthine)^3^. In human cells, DNA base damage is primarily detected by damage-specific DNA glycosylases^4,5^. DNA glycosylase recognizes damaged base and catalyzes its excision, leaving an apurinic/apyrimidinic site (AP site) which will be cleaved by AP site endonuclease APE1 to generate a nick on DNA^6^. Subsequent repair reactions involve AP-lyase activity, DNA polymerase activity and DNA ligase activity. These reactions are executed by XRCC1, DNA polymerase β and DNA ligase III in the short-patch BER sub-pathway, while the long-patch BER sub-pathway requires the replicative DNA polymerase δ/ε-PCNA-RFC machinery, flap endonuclease 1 and DNA ligase I^7^.

Alkyladenine DNA glycosylase (AAG, 3-methyladenine DNA glycosylase), as one of the first responders of DNA base damage, can recognize alkylpurines like 3-methyladenine (3-MA) and 7-methylguanine (m^7^G)^8,9^, oxidized adenine 1,*N*^6^-ethenoadenine (εA)^10^ and deaminated adenine hypoxanthine^6,11–13^. In humans, altered expression and single nucleotide polymorphisms of AAG are associated with microsatellite instability^14^, spontaneous frameshift mutagenesis^15^, and a variety of cancers including osteosarcoma^16^, breast cancer^17,18^ and astrocytic tumors^19,20^. In mouse models, Aag-knockout mice are prone to liver and colorectal cancers^21,22^, while excessive AAG activity in Aag-overexpressing mice causes hepatotoxicity, lethality and other alkylation-induced toxicity^23,24^.

Frequently occurring DNA base damages could be present in all regions of chromatinized eukaryotic genome, including nucleosomal DNA sites where nucleosomes would obstruct BER machinery’s accessibility to the damage sites^25^. In a nucleosome, the 147-bp DNA wraps around the octameric core in 1.65 superhelical turns, leaving only a portion of solvent-facing DNA freely accessible^26,27^. Extensive in vitro studies showed that AAG^28,29^ along with short-patch BER factors^30–32^ could selectively locate and bind to the damage sites in nucleosomes. In general, AAG activity on nucleosome is strongly correlated to solution accessibility of the damaged base, and in vivo studies also confirmed this general correlation^33^, and found that bases at the DNA minor grooves, which interact with the octameric histone core, display higher mutation rates due to low accessibility to repair factors^25^. Structurally, the solution accessibility is predominantly determined by its translational position and rotational orientation on a nucleosome, and solvent-facing damaged bases were indeed excised more efficiently than occluded and embedded damaged bases with medium and low solution accessibility^34^. Another contributing factor is the structural dynamics of nucleosome such as spontaneous opening of nucleosome^35,36^, which could impact on the accessibility of the damaged bases on certain positions. In addition, compared to the other regions of the nucleosomal DNA, the dyad axis is the least accessible to repair factors^34,37^. Overall, the differential AAG activity at various nucleosomal positions could not be solely explained by the solution accessibility predicted from a static nucleosome structure.

While the structural mechanism of base excision enzymes’ action on naked DNA duplex is well-understood^4,38–43^, how these proteins overcome nucleosome-imposed obstacle to locate and repair DNA base damage in nucleosome is not fully understood. In the present study, we employed cryo-EM to explain how the BER factor AAG accesses a damaged base introduced to various positions on nucleosomes. Our results show that a single DI nucleotide could induce a global perturbation to the nucleosome structure, and AAG exploits altered nucleosome dynamics and adopts distinct mechanisms to access the substrate site dependent on the geometric positions of the damaged base on the nucleosome.

## Results

### Preparation and structural determination of the AAG–NCP complexes

**Fig. 1:**
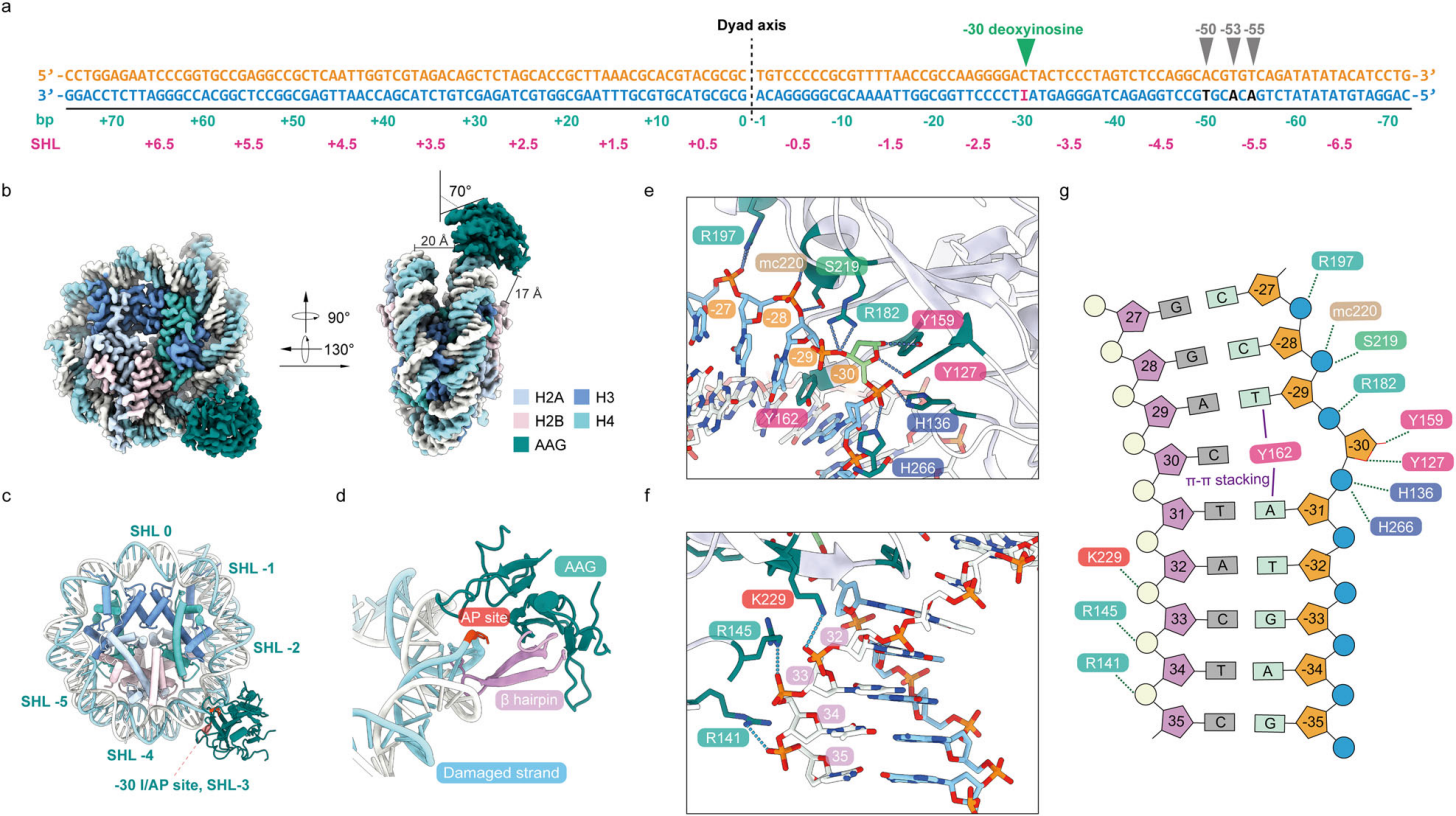
Cryo-EM structure of the AAG–NCP^–30AP^ complex. **a** Widom 601 sequence bearing DI in various positions (–30, –50, –53 and –55). **b** Cryo-EM structure of the DNA glycosylase AAG in complex with an NCP bearing DI/AP site at –30 position, designated as AAG–NCP^–30AP^ complex. **c** Atomic model of AAG–NCP^–30AP^ complex. **d** A protruding β-hairpin (β3–β4) of AAG is inserted into the minor groove of the nucleosomal DNA. **e** Close-up view of the contacts between AAG and the DI/AP site-containing damaged strand of the nucleosomal DNA. **f** Close-up view of the contacts between AAG and the undamaged strand of the nucleosomal DNA. **g** Schematic representation of the atomic contacts in AAG–NCP^–30AP^ complex.

To capture the state of DNA glycosylase AAG engaging with DNA base damage in nucleosome, we first assembled nucleosome core particles (NCPs) from a 152-base pair (bp) Widom 601 DNA^44^ and core histones H2A, H2B, H3 and H4 from *Xenopus laevis* (Supplementary Fig. S1a)^45–47^. The bottom strand of Widom 601 DNA (designated as damaged strand) contains a DI to mimic a base damage resulting from deamination of DA (Fig. 1a).

To investigate how AAG acts in positions with different solution accessibility and histone microenvironment, we sampled different superhelical locations (SHLs) and rotational orientations on nucleosome, and a series of single DI-containing DNAs with a DI located in various geometric positions (–30, –50, –53 and –55) were prepared (Fig. 1a). –30 at SHL-3 and –50 at SHL-5 both represent solvent-facing positions with high solution accessibility, whereas –53 and –55 at SHL-5 represent occluded and embedded positions with medium and low solution accessibility, respectively^34^. We assembled these NCPs and incubated them with purified AAG to form AAG–NCP complexes, which were further analyzed by cryo-EM (Supplementary Figs. S2–S5).

### Overall structure of the AAG–NCP^–30AP^ complex with a fully exposed damage site

The presence of DI in the –30 position of nucleosomal DNA (NCP^–30I^), which is solvent-facing, triggers a strong engagement of AAG, as shown in the glycerol density gradient centrifugation (Supplementary Fig. S1b, c). Cryo-EM analysis of the AAG–NCP^–30I^ sample obtained high-resolution structures of the NCP in both AAG-bound and -unbound states, at resolutions of 2.9 Å and 2.8 Å, respectively (Supplementary Fig. S2). The nucleosomal DNA in the two states and most of the side chains of AAG in the bound state are well resolved (Supplementary Fig. S6a–d). In the map of the free NCP (NCP^–30I^), the DI in the –30 position is well resolved and forms a non-canonical base pair with the DC on the top strand (Supplementary Fig. S6a), reflecting a state for the damaged nucleosome before AAG binding. In contrast, the map of the AAG-bound NCP (AAG–NCP^–30AP^) was identified as a post-catalytic state. The ribose ring of the –30 position is in a flipped-out orientation, and there is no density for hypoxanthine base at –30, indicating that this is a post-catalytic AP site (Supplementary Fig. S6b). In addition, superimposition of a DI onto the catalytic cave comprising residues Y159, Y127, L180, R182, E125 and C167 would result in multiple steric clashes (Supplementary Fig. S6e).

This high-resolution structure of the AAG–NCP^–30AP^ complex provides an accurate model explaining the molecular details of the interactions between the damaged NCP and AAG. In general, AAG attaches to the minor groove of the nucleosomal DNA at SHL-3 and is angled at ~70° against the plane parallel to the nucleosomal disc (Fig. 1b, c). The distance between AAG and the closest histone is beyond 17 Å, and no interactions between AAG and octameric histone core were observed (Fig. 1b). In addition to the protruding β-hairpin of AAG, which is inserted into the minor groove of nucleosomal DNA for direct damage recognition (Fig. 1d), a few other residues also contribute to the stabilization of AAG on the nucleosome. On the damaged strand, R197, S219 and the main chain of K220 display a direct interaction with the DNA backbone (Fig. 1e, g and Supplementary Fig. S6c, d). On the undamaged strand, the attachment of AAG to NCP is strengthened by the interactions between the phosphate backbone and three successive positively charged residues R141, R145 and K229 (Fig. 1f, g and Supplementary Fig. S6c, d).

### Interaction between AAG and the AP site in the AAG–NCP^–30AP^ complex

**Fig. 2:**
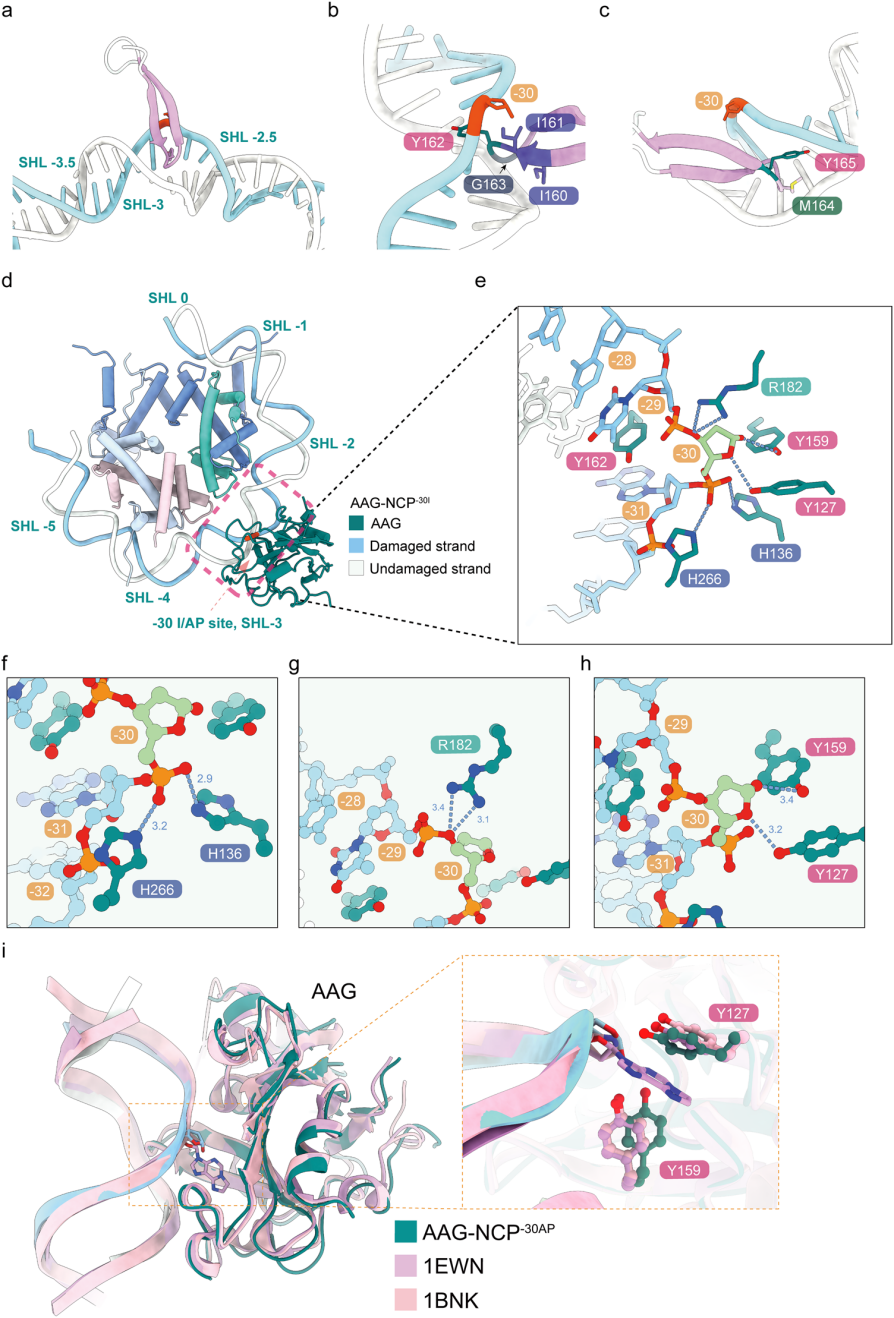
Mechanism of DNA base damage recognition by AAG in the nucleosome. **a**–**c** AAG identifies the damaged base by inserting the β3–β4 hairpin into the damage-located minor groove, and displaces the damaged base using Y162. **d**, **e** Overview of interactions between the flipped post-catalytic AP site and AAG residues (Y127, H136, Y159, R182, H266). **f**–**h** Close-up views of the detailed interactions between the post-catalytic AP site and AAG. **i** Structural comparison of AAG–NCP^–30AP^, E125Q AAG/εA-DNA complex (PDB: 1EWN) and AAG complexed to DNA containing a pyrrolidine abasic nucleotide (PDB: 1BNK). Zoom-in view shows the local conformational variations among three structures.

In the AAG–NCP^–30AP^ structure, AAG shadows ~8 bp of nucleosomal DNA near SHL-3 (Fig. 1g). The inserted β-hairpin loop is composed of IIYGMY (residues 160–165), which makes extensive contacts with the skewed DNA and serves as the damage-recognition motif (Figs. 1d, 2a and Supplementary Fig. S7a). The residues I160 and I161 establish hydrophobic interactions with both sides of DNA in the minor groove (Fig. 2b). The aromatic residue Y162 stacks with the bases of –31A and –29T on the damaged strand through π–π interactions. Bridging residue G163 bents the terminal loop and links the β3 and β4 of AAG (Fig. 2b). The main chain of G163 also contributes to the stabilization of the β-hairpin by interacting with 31T on the undamaged strand. Scaffolding residues M164 and Y165 locate in the center of the minor groove and widen the distance between two DNA backbones (Fig. 2c).

The flipped AP site is stabilized through three sets of interactions (Figs. 1g, 2d, e) and the interacting residues of AAG are highly conserved (Supplementary Fig. S8). The first interacting site consists of two basic amino acids H136 and H266 that coordinate the phosphate backbone of the AP site (Fig. 2e, f). These two histidine residues interact with the phosphate group between –30 and –31 to keep it in a proper conformation. The second site involves a positively charged residue R182 that forms a hydrogen bond with the O3′ atom of the AP site (Fig. 2e, g). The third site involves a direct interaction of the AP-site ribose ring with two catalytically important residues, Y127 and Y159 (Fig. 2e, h). Comparison of the AAG–NCP^–30AP^ structure with the crystal structure of AAG bound to pyrrolidine-containing free duplex DNA (PDB: 1BNK)^39^ revealed a different binding mode for these two residues (Fig. 2i). In the AAG–NCP^–30AP^ structure, Y127 and Y159 interact with the AP site through hydrogen bonds (Fig. 2e, h), whereas the interaction between Y159 and the AP site is absent in the previously reported crystal structure, likely due to the lack of a hydroxyl group in pyrrolidine. In the crystal structure of AAG–DNA complex containing a 1,*N*^6^-ethenoadenine in the pre-catalysis state (PDB: 1EWN)^48^, Y127 and Y159 sandwich the flipped base, and Y127 directly stacks with the modified base (Fig. 2i). Notably, most of these AP site-interacting residues are invariant through evolution (Supplementary Fig. S8), and a previous site-directed random mutagenesis study showed that Y127 could not be substituted and that Y159 was among the group with the lowest mutability^49^.

Overall, AAG uses a similar set of conserved residues to interact with the DNA substrate in both the linear and nucleosomal forms.

### Distortion of nucleosomal DNA in the NCP^–30I^ and AAG–NCP^–30AP^ structures

**Fig. 3:**
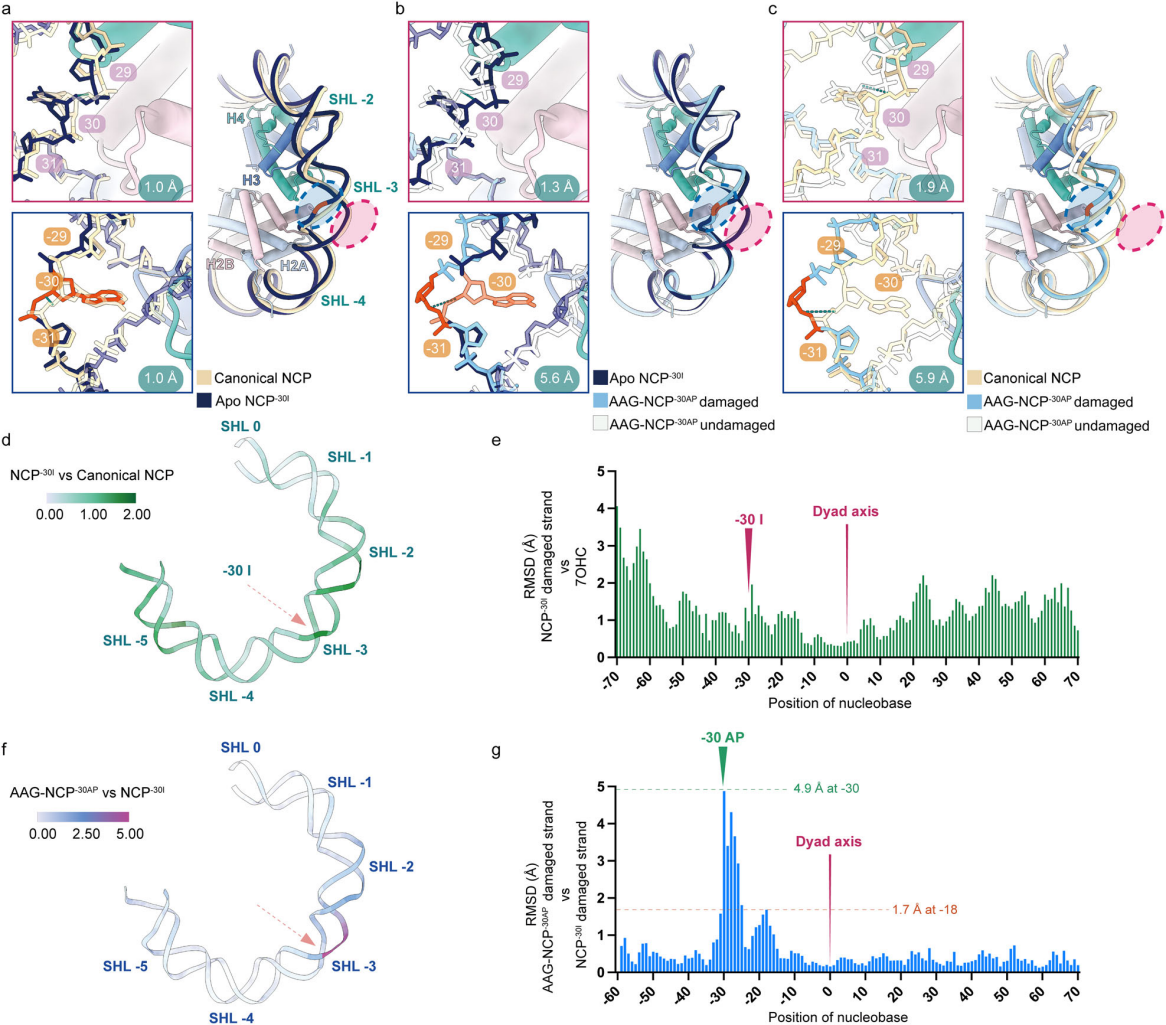
DNA distortion in NCP^–30I^ and AAG–NCP^–30AP^ structures. **a** Nucleosomal DNA deformation in the NCP^–30I^ complex in comparison with a canonical NCP (PDB: 7OHC). Undamaged strand is labeled with red circle (right panel) and rectangle (upper left panel), and RMSD of DNA backbone from 25 to 35 is 1.0 Å. Damaged strand is labeled with blue circle (right panel) and rectangle (lower left panel), and RMSD of DNA backbone from –35 to –25 is 1.0 Å. **b** Local DNA distortion of the AAG–NCP^–30AP^ complex in comparison with NCP^–30I^. RMSDs are 1.3 Å and 5.6 Å for undamaged and damaged strands, respectively. **c** The collective nucleosomal DNA deformation of the AAG–NCP^–30AP^ in comparison with a canonical NCP. RMSDs are 1.9 Å and 5.9 Å for undamaged and damaged strands, reepectively. **d**, **e** Temperature map and RMSD–residue plot representing DI-caused perturbation of the nucleosomal DNA calculated by comparing the NCP^–30I^ to the canonical NCP. **f**, **g** Temperature map and RMSD–residue plot representing the AAG-imposed local DNA distortion calculated by comparing the AAG–NCP^–30AP^ to the NCP^–30I^. Note that the terminal DNA (from –60 to exit) of the AAG–NCP^–30AP^ is relatively flexible in the map and not modeled, and therefore panel (**g**) only includes information from –59 to 72.

Importantly, the structures of the NCP^–30I^ and AAG–NCP^–30AP^ demonstrate apparent differences in conformation of nucleosomal DNA when compared with the structures of three representative canonical nucleosomes (PDB: 7OHC, 6ZHX, 6WZ5)^50–52^. These nucleosomes were all derived from *X. laevis* histones and the Widom 601 DNA sequence. The voxel sizes of the published maps were calibrated and the corresponding models were refined in real space before comparison. In the NCP^–30I^, the hypoxanthine base of DI pairs to pyrimidine through weaker hydrogen bonds compared to Watson-Crick base pairs, which destabilizes local DNA and leads to a global perturbation of nucleosomal DNA (Fig. 3a, d, e and Supplementary Fig. S9). Specifically, compared to the root-mean-square deviation (RMSD) values between two canonical nucleosomal DNA backbones (Supplementary Fig. S9c–e), the RMSD values between the damaged-strand DNA of apo NCP^–30I^ and that of the canonical NCP are much larger, and increasing displacements are shown towards the nucleosomal DNA ends, especially at the end close to the DI (Fig. 3d, e and Supplementary Fig. S9a, b). Locally, this perturbation is characterized by an outward movement of the DNA from the histone core: the calculated RMSD of the DNA backbone from –25 to –35 (–30 ± 5 positions) is 1.0 Å for both the damaged and intact strands (Fig. 3a).

In the AAG–NCP^–30AP^, the engagement of AAG further distorts the nucleosomal DNA. Previous studies on free DNA substrate indicated that glycosylases require DNA bending at a certain angle to ensure base-flipping and optimal catalysis^53^. Comparing with the NCP^–30I^ structure, additional bending and twisting of the nucleosomal DNA around the damage site were observed, which might lead to an optimal DNA bending angle for AAG engagement (Fig. 3b, f, g). In general, binding of AAG led to further outward displacement of the DNA around SHL-3, and this distortion was local compared to the global perturbation caused by DI in the NCP^–30I^ (Fig. 3f). The distortion out of SHL-3 rapidly decreased, and the maximum was observed at –30 with an RMSD of 4.9 Å (Fig. 3g). In addition, a second distortion peak was seen to be centered around –18 (SHL-2) position (Fig. 3g). The calculated RMSDs around the –30 AP site from –35 to –25 (–30 ± 5 positions) are 5.6 Å and 1.3 Å for the damaged and undamaged strands, respectively (Fig. 3b). A direct comparison of AAG–NCP^–30AP^ structure to the canonical NCP confirmed a very dramatic displacement of the damaged strand near SHL-3 (RMSD: 5.9 Å) (Fig. 3c).

Nucleosome imposes restriction to DNA through interactions between nucleosomal DNA and histones^54^. Histone core makes contacts with DNA backbone when the minor groove of nucleosomal DNA faces histone core. Therefore, we analyzed the changes of the interactions between the histone core and the DNA backbone. The presence of DI in NCP^–30I^ causes a reduction of the buried surface area from 7141 Å^2^ to 6195 Å^2^, indicating that DI alone has significantly destabilized the interaction between the nucleosomal DNA and the octameric histone core (Supplementary Table S1). Further engagement of AAG has a marginal effect on the overall buried surface area (6166 Å^2^), since the dramatic DNA distortion is limited to local region around the damaged site (Fig. 3b).

These results suggest that the damaged base-induced global perturbation of nucleosomal DNA destabilizes the nucleosome and relives nucleosome-imposed restriction, which likely plays a determinant role in AAG recruitment. Due to the high solution accessibility of the –30 position, additional local DNA distortion induced by AAG alone is sufficient to deform nucleosomal DNA and allow AAG engagement to the damaged site.

### Distortion of nucleosomal DNA in the NCP^–50I^ and AAG–NCP^–50AP^ structures

**Fig. 4:**
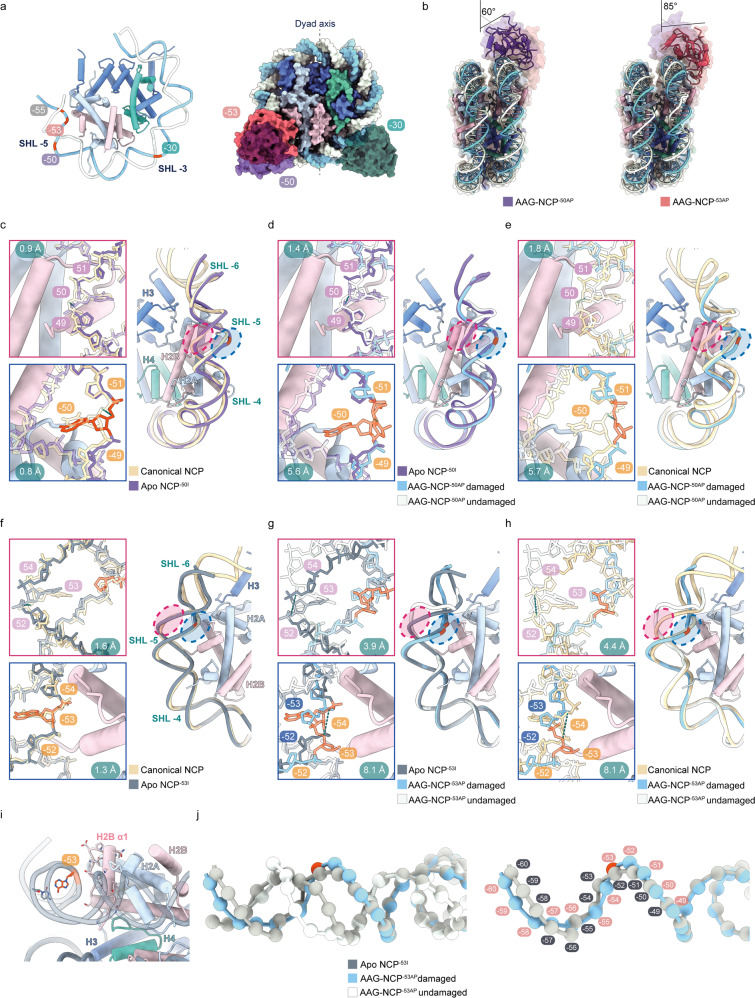
Structural comparison between NCP^–50I^, AAG–NCP^–50AP^, NCP^–53I^, AAG–NCP^–53AP^ and the canonical NCP. **a** Superimposition of the models of the AAG–NCP^–30AP^, AAG–NCP^–50AP^ and AAG–NCP^–53AP^ to highlight the orientation difference of AAG in the three structures. **b** Comparison of the AAG–NCP^–50AP^ and AAG–NCP^–53AP^. **c** The nucleosomal DNA perturbation in the NCP^–50I^ in comparison with a canonical NCP (PDB: 7OHC). RMSD of the undamaged DNA backbone from 45 to 55 is 0.9 Å, and RMSD of the undamaged strand from –55 to –45 is 0.8 Å. **d** Local DNA distortion of the AAG–NCP^–50AP^ in comparison with the NCP^–50I^. RMSDs are 1.4 Å and 5.6 Å for the undamaged and damaged strands, respectively. **e** The nucleosomal DNA deformation of the AAG–NCP^–50AP^ in comparison with a canonical NCP. RMSDs are 1.8 Å and 5.7 Å for the undamaged and damaged strands, respectively. **f** The nucleosomal DNA perturbation in the NCP^–53I^ in comparison with a canonical NCP (PDB: 7OHC). RMSD of the undamaged DNA backbone from 48 to 58 is 1.6 Å, and RMSD of the undamaged strand from –58 to –48 is 1.3 Å. **g** In the AAG–NCP^–53AP^, drastic local DNA distortion is accompanied by local register shift. In comparison with the NCP^–53I^, RMSD of the undamaged strand is 3.9 Å, and the RMSD of the damaged strand is 8.1 Å. AP site at –53 is relocated to –52 in relative to the register of the NCP^–53I^. **h** The nucleosomal DNA deformation in the AAG–NCP^–53AP^ in comparison with a canonical NCP^–53I^. RMSDs are 4.4 Å and 8.1 Å for the undamaged and damaged strands, respectively. **i** The original DNA register of the NCP^–53I^ prohibits base flipping at –53, due to steric clash with histone H2B. **j** The local register shift of the AAG–NCP^–53AP^ in comparison to the register of the NCP^–53I^.

We next examined nucleosomes with a damaged base at a different SHL (SHL-5) to study the effect of translational position. In the AAG–NCP^–50AP^ complex, a DI was introduced into the position of –50 at SHL-5, which could be regarded as a roughly equivalent position of –30 in terms of solution accessibility (Fig. 4a, b). Subsequently, the structures of apo NCP^–50I^ and AAG–NCP^–50AP^ complexes were solved at resolutions of 2.9 Å and 3.0 Å, respectively (Supplementary Figs. S3, S7, S10a, b). Despite the overall high resolution of the AAG–NCP^–50AP^ structure, the portion of AAG becomes highly fragmented in the sharpened map, indicating a conformational wobbling of AAG on the nucleosome. Nevertheless, the nucleosomal DNA including the AP site and the inserted residue Tyr162 are well resolved (Supplementary Fig. S10b), enabling the modeling of the DNA and the assignment of its post-catalytic state. The overall position of AAG was determined by rigid-body fitting.

Comparison of the apo NCP^–50I^ to the canonical NCP (PDB: 7OHC)^50^ reveals a similar pattern of global perturbation as seen in the structure of the NCP^–30I^ (Supplementary Fig. S11a). The presence of DI at –50 causes a noticeable movement of both strands away from the histone core: at –55 to –45 (–50 ± 5 positions) of SHL-5, the calculated RMSDs for the DNA backbone are 0.8 Å and 0.9 Å for the damaged and undamaged strands, respectively (Fig. 4c).

In the AAG–NCP^–50AP^ structure, the engagement of AAG is similar to that in the AAG–NCP^–30AP^ in general, although AAG in AAG–NCP^–50AP^ is angled at ~60° against the plane parallel to the nucleosome disc (Fig. 4b). Globally, the buried surface area between the DNA and the histone core decreases from 7134 Å^2^ to 6248 Å^2^ in the presence of DI at –50, and further decreases to 5977 Å^2^ after engaging with AAG (Supplementary Table S1). Further DNA distortion was also observed upon AAG engagement, and displayed a predominantly local effect: the maximal distortion was observed at –50 with an RMSD of 3.0 Å (Supplementary Fig. S11b), and the average displacements for the damaged and undamaged strands at –55 to –45 of SHL-5 (–50 ± 5 positions) were 5.6 Å and 1.4 Å, respectively (Fig. 4d). When a canonical NCP was used for comparison, the DNA backbone RMSDs at SHL-5 were 5.7 Å and 1.8 Å for the damaged and undamaged strands, respectively (Fig. 4e).

The degree of local DNA distortion in the AAG–NCP^–50AP^ is less than that in the AAG–NCP^–30AP^, but the overall similarity suggests a general mechanism of the engagement of AAG with DNA base damage in solvent-facing positions, which leverages on the global structural perturbation of the nucleosome by DI nucleotide. Since the –50 position is closer to one end of the DNA than the –30 position, the large local DNA distortion around the damage site could have been relaxed by propagating to the end.

### Structure of the AAG–NCP^–53AP^ complex containing an occluded damaged base with medium solution accessibility

Within an SHL region of the nucleosomal DNA, damaged bases in different rotational orientations should have sharply different solution accessibility, and thus different accessibility to repair proteins. Although the DI in the NCP^–53I^ is at an occluded position of SHL-5 with medium solution accessibility (Figs. 1a, 4a), our data show that the AAG–NCP^–53^ complex displays a comparable high assembly efficiency as the AAG–NCP^–30^ and AAG–NCP^–50^ complexes (Supplementary Fig. S4a). We subsequently determined the structures of the NCP^–53I^ and AAG–NCP^–53AP^ at resolutions of 2.8 Å and 3.1 Å, respectively (Supplementary Figs. S4, S7, S10c, d). Similar to the AAG–NCP^–50AP^ complex, AAG in AAG–NCP^–53AP^ is flexible, but the nucleosomal DNA including the AP site and the inserted residue Tyr162 are nicely resolved (Supplementary Fig. S10d). Structural comparison of the NCP^–53I^ to the canonical NCP (PDB: 7OHC) reveals a similar global perturbation as in the NCP^–30I^ and NCP^–50I^ (Supplementary Fig. S11c), featuring a local outward movement of the nucleosomal DNA around the damage site, with the calculated RMSDs of the DNA backbone for the damaged and undamaged strands (–53 ± 5 positions) being 1.3 Å and 1.6 Å, respectively (Fig. 4f).

AAG in AAG–NCP^–53AP^ is angled at ~85° against the plane parallel to the nucleosome disc (Fig. 4b). Importantly, in the AAG–NCP^–53AP^, a drastic local DNA distortion was observed (Fig. 4g, h), and the maximal distortion is at –53 with an RMSD of 7.4 Å (Supplementary Fig. S11d). There is a significant widening of the minor groove in the local region of –53 position compared to the apo NCP^–53I^ (Fig. 4j), which is also reflected by much larger displacements for both the damaged and undamaged strands: the calculated RMSDs of the DNA backbones from –58 to –48 (–53 ± 5 positions) are 8.1 Å and 3.9 Å for the damaged and undamaged strands, respectively (Fig. 4g). When comparing the AAG–NCP^–53AP^ with a canonical NCP (PDB: 7OHC), the RMSDs for the two strands are 8.1 Å and 4.4 Å, respectively (Fig. 4h). Globally, the buried surface area between the DNA and the histone core decreases from 7141 Å^2^ to 6075 Å^2^ in the presence of DI at –53, and further decreases to 5897 Å^2^ after engaging with AAG (Supplementary Table S1).

Intriguingly, in comparison to the local DNA distortions in the AAG–NCP^–30AP^ and AAG–NCP^–50AP^ (Supplementary Videos S1, S2), an additional translocation of local DNA around –53 was observed (Supplementary Videos S3, S4). The AP site at –53 in the AAG–NCP^–53AP^ is relocated to –52 in spatial relation to the translational register of NCP^–53I^. Since base-flipping at –53 on a regular NCP would cause a clash with H2B, this local register shift from –53 to –52 appears to be necessary for AAG to interact with the flipped base (Fig. 4i). It must be noted that the global translational register is not affected, and this translocation is restricted to the region from the damage site to the near exit end of the nucleosomal DNA.

Therefore, these observations are in line with the previous in vitro data that when the εA lesion was placed in –126 position (–53 in our structure), AAG was still highly active at this position^34^, indicating that for positions with medium solution accessibility, AAG could induce drastic local DNA distortion that allows local register shift to increase the solution accessibility of the occluded damaged base (Fig. 4j).

### Partial opening of the nucleosome in the AAG–NCP^–55^ complex

**Fig. 5:**
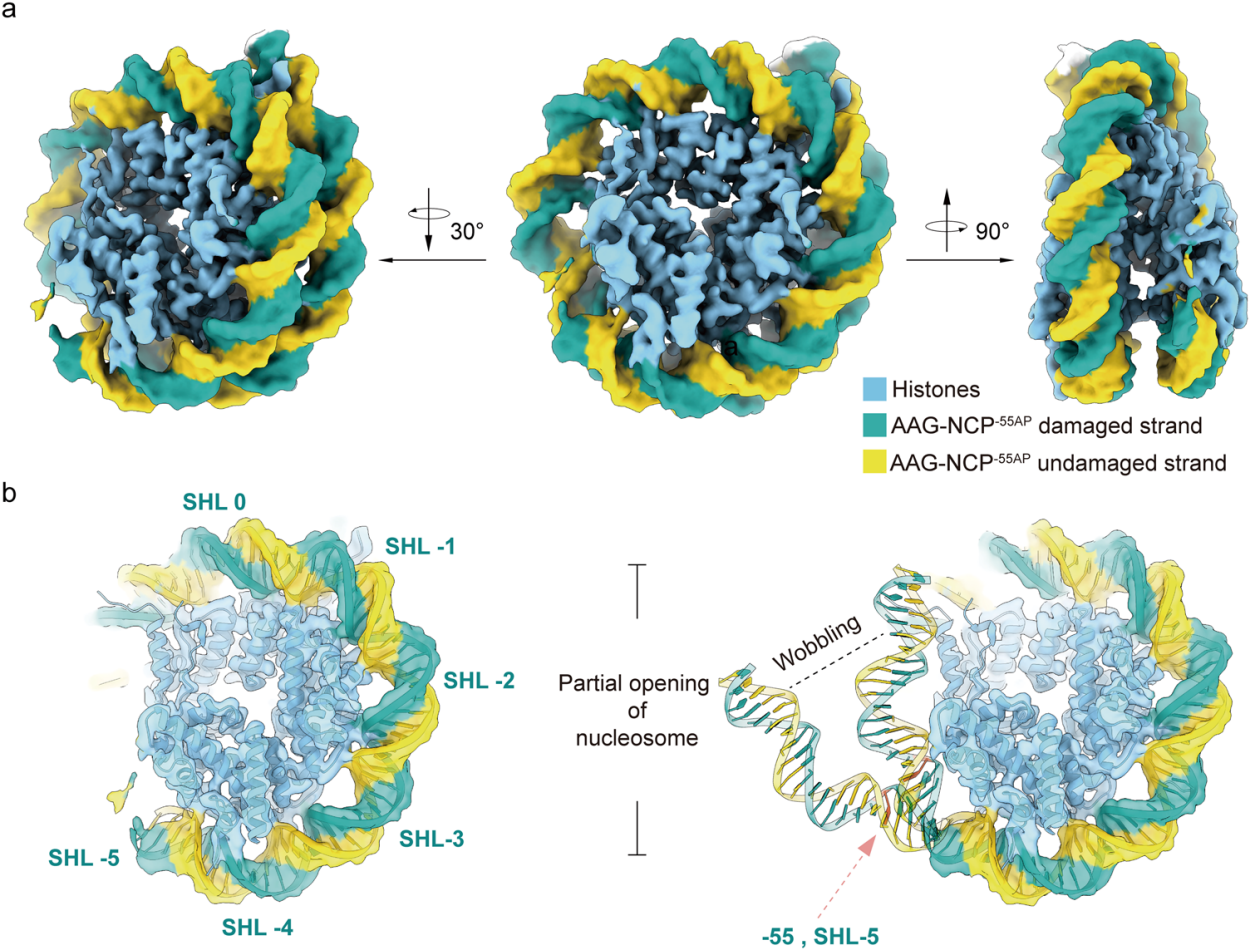
Partial opening of nucleosome in the AAG–NCP^–55AP^. **a** Cryo-EM map of the AAG–NCP^–55AP^ complex. **b** Partial opening of nucleosome in the AAG–NCP^–55AP^. DNA from SHL-5 to the proximal end is peeled off from the histone core.

Different from the previous DI-containing NCPs, the DI in the AAG–NCP^–55AP^ is in a completely buried position with low solution accessibility. Therefore, one would expect a very low activity of AAG at this site. To our surprise, the NCP^–55I^ is still capable of forming a stable complex with AAG, as shown in the glycerol density gradient experiment (Supplementary Fig. S5a). Similarly, cryo-EM analysis revealed two major conformational populations for the AAG–NCP^–55^ sample. One is the structure of NCP^–55I^ determined at 2.8 Å (Supplementary Figs. S5, S7, S10e), and the other is an unusual NCP structure at 2.9 Å resolution, with the terminal nucleosomal DNA highly flexible, unresolved in the map (from SHL-5 to the proximal end) (Fig. 5a). In this unusual structure, however, density of AAG could not be found. Given the strong association of AAG with the NCPs in our biochemical preparation (Supplementary Fig. S5), it is highly likely that this structure in fact reflects the AAG–NCP^–55AP^ complex, in which a drastic conformational change of the DNA at the region of –55 has caused the opening of the terminal DNA (Fig. 5b). This assignment is also supported by the previous data that AAG still retained ~50% activity towards a damaged base at –55 position^34^.

Similarly, a global perturbation of nucleosomal DNA by DI in NCP^–55I^ was observed, but the proximal end of the DNA displays a much larger displacement (Supplementary Fig. S11e). The buried surface area between the DNA and the histone core decreases from 7517 Å^2^ to 6745 Å^2^ in the presence of DI at –55 (Supplementary Table S1). Locally at –60 to –50 (–55 ± 5 positions), the –55 DI-induced structural displacements of DNA backbone are 1.1 Å and 1.3 Å for the damaged and undamaged strands, respectively, which are in a similar range to those observed in the NCP^–30I^, NCP^–50I^ and NCP^–53I^ complexes (Supplementary Fig. S12).

It is of low possibility that this unusual NCP is still in the pre-catalytic state before AAG binding. The dynamic nature of nucleosome could lead to spontaneous unwrapping of nucleosomal DNA^35–37^, and the significant destabilization of the DNA at the exit observed in the NCP^–55I^ could further increases the unwrapping possibility. In this scenario, the partial opening of nucleosomal DNA gives a short-lived window for AAG to access the damaged site. The binding of AAG to the detached DNA duplex renders this process less reversible, allowing the formation of a stable AAG–NCP^–55^ complex for subsequent excision reaction.

Altogether, the presence of a DI in a completely buried position also leads to a global perturbation of the nucleosomal DNA. When the damaged base is near the exit end of the nucleosomal DNA, the perturbation could increase the possibility of terminal DNA duplex opening to facilitate AAG engagement.

## Discussion

Over the past several decades, immense amount of effort has been devoted to the elucidation of principles governing interactions between DNA-binding proteins and free DNA duplex (reviewed in^55,56^). However, in a more physiological context, how DNA-binding proteins interrogate nucleosomes to detect and read hidden information on chromatin is still not completely clear^57^. Recent structural studies on nucleotide excision repair^58^, retroviral integration^59,60^ and pioneer transcription factors^61,62^ have revealed that these factors could employ a few different mechanisms, such as nucleosomal DNA register shifting and local DNA distortion, to induce nucleosome deformation to fulfill their molecular functions.

In the present work, we determined a set of cryo-EM structures of the NCPs and AAG–NCP complexes with a damage-mimicking DI nucleotide placed in different representative positions on nucleosome. Although AAG proteins in these complexes are positioned differently on the nucleosomes, the binding mode of AAG on the nucleosome is generally similar to that on free DNA duplex^39,48^, with only limited interactions through several evolutionarily invariant residues with the negatively charged DNA backbone^49^ (Fig. 1g). These structures also show a highly conserved mechanism for AAG to recognize the damaged base, involving the insertion of a protruding β-hairpin into the minor groove of the damage site, the replacement of the damaged base by a tyrosine residue, and stabilization of the flipped base/AP site with two aromatic residues (Figs. 1d, 2a–h). These similarities suggest that a crucial and potentially rate-limiting step for base excision reactions could be the recruitment of AAG onto the damage sites.

Interestingly, with pair-wise comparison to the canonical NCP, we discovered that the presence of a single DI nucleotide alone is sufficient to perturb nucleosomal DNA globally (Fig. 3d, e and Supplementary Fig. S11a, c, e), resulting in a collectively reduced buried surface area between nucleosomal DNA and histone core, by 10%–15% reduction depending on the DI position (Supplementary Table S1). These global perturbations of nucleosomal DNA are generally in a similar pattern regardless of the position of the damaged base, and the DNA deformation caused by DI is most apparent near the exit of nucleosomal DNA (Fig. 3d, e and Supplementary Fig. S11a, c, e).

After formation of the stable AAG–NCP^AP^ complex, the binding of AAG induces a dramatic but relatively local distortion on nucleosomal DNA around the damaged base (Fig. 3f, g and Supplementary Fig. S11b, d). Depending on the translational and rotational positions of the damaged site, AAG makes use of distinct mechanisms to get access to the damaged base. For the DI in solvent-facing positions with high solution accessibility, where AAG is highly active^34^ and has a direct access to these positions, AAG directly augments the local DNA distortion to recognize the damage site (Supplementary Fig. S13a). For the DI in occluded positions such as –53 with medium accessibility, where AAG activity is expected to be lower comparing to solvent-facing positions^34^, AAG induces drastic local DNA distortion, including both twisting and translocation of DNA to generate a shift of local DNA register by 1 bp to relieve the nucleosome-imposed spatial hindrance for accessing the damaged site (Fig. 4i, j and Supplementary Fig. S13b). As for deeply embedded positions (e.g., –55 in our study), local DNA distortion and limited register shift are insufficient to fully expose the buried base. Our results suggest that globally perturbed nucleosome by DI might be more prone to spontaneous unwrapping^37^. AAG can make use of this feature to capture the detached terminal DNA duplex and render this process less reversible to favor the unwrapping direction. Therefore, in these completely buried sites, a partial opening of the nucleosomal DNA might be a prerequisite for the recruitment and catalysis of AAG (Fig. 5b and Supplementary Fig. S13c).

Altogether, in all these above-mentioned scenarios, the altered structural dynamics of the nucleosome likely plays a major role in recruiting AAG to the damaged sites. The global perturbation of the nucleosomal DNA by DI nucleotide simply reflects the fact that disruption of a single base pair could weaken DNA–histone interaction and alter the conformational landscape of the nucleosome. In a kinetics view, this would result in an increased sampling of the otherwise less possible conformations of the nucleosome. AAG is capable of capturing these transient conformations and forms a stable AAG–NCP complex for the subsequent excision reaction. In summary, our work reveals the effect of a damaged base on nucleosome stability and provides a mechanistic framework for understanding how the DNA glycosylase AAG exploits the structural dynamics of nucleosome to engage with DNA base damage in the nucleosome. In a broader context, our work also contributes to the general knowledge of how the structural dynamics of nucleosome interplays with DNA-binding proteins to regulate their actions on chromatinized eukaryotic genome.

## Materials and methods

### Construct design and protein expression

The gene encoding the N-terminus-truncated sequence of human AAG (residues 80–298) was cloned into pET22b vector with an N-terminal 6× His tag. Plasmid verified by sequencing was transformed into BL21 (DE3) *Escherichia coli* cells for overexpression. At OD_600_ of 0.6, expression of AAG was induced by 0.1 mM isopropyl β-D-1-thiogalactopyranoside (IPTG) at 18 °C for 17 h. Cells were collected by centrifugation (3500 rpm, 10 min, 25 °C) and resuspended in buffer A (20 mM Tris-HCl, pH 7.5, 100 mM NaCl). The suspension was flash-frozen in liquid nitrogen and stored at –80 °C for purification.

### Purification of AAG

All steps were carried out at 4 °C. Frozen cells were thawed and lysed by sonication in buffer A supplemented with 0.1 mM phenylmethanesulfonyl fluoride. The lysate was then supplemented with 0.5 U/mL benzonase nuclease and centrifuged at 18,000 rpm for 40 min. The supernatant was filtered with a 0.45-μm syringe filter, then loaded onto a 5-mL Ni-smart beads 6FF FPLC column (Smart-Lifesciences, Changzhou), and eluted with a linear gradient of imidazole using buffer A and buffer B (20 mM Tris-HCl, pH 7.5, 100 mM NaCl, 1 M Imidazole). Peak fractions were collected and pooled for dialysis in buffer C (20 mM Tris-HCl, pH 7.5, 100 mM NaCl, 1 mM EDTA, 1 mM DTT) overnight to remove imidazole. Dialyzed sample was centrifuged to remove any possible precipitates, and the supernatant was loaded onto a 5-mL Heparin beads 6FF FPLC column (Smart-Lifesciences, Changzhou) pre-equilibrated with buffer C, and then eluted with a linear gradient of buffer C and buffer D (20 mM Tris-HCl, pH 7.5, 1 M NaCl, 1 mM EDTA, 1 mM DTT). Peak fractions were pooled and concentrated, and then loaded onto a Superdex 75 10/300 GL (Cytiva) column pre-equilibrated in buffer C. Peak fractions were collected and analyzed by SDS-PAGE. The AAG proteins were concentrated to 1 mg/mL and stored at –80 °C in small aliquots.

### Widom 601 DNA preparation

#### Widom 601 top strand sequence

CCTGGAGAATCCCGGTGCCGAGGCCGCTCAATTGGTCGTAGACAGCTCTAGCACCGCTTAAACGCACGTACGCGCTGTCCCCCGCGTTTTAACCGCCAAGGGGATTACTCCCTAGTCTCCAGGCACGTGTCAGATATATACATCCTGTGCAT

#### Widom 601 –30I bottom strand sequence

ATGCACAGGATGTATATATCTGACACGTGCCTGGAGACTAGGGAGTA**I**TCCCCTTGGCGGTTAAAACGCGGGGGACAGCGCGTACGTGCGTTTAAGCGGTGCTAGAGCTGTCTACGACCAATTGAGCGGCCTCGGCACCGGGATTCTCCAGG

#### Widom 601 –50I bottom strand sequence

ATGCACAGGATGTATATATCTGACACG**I**GCCTGGAGACTAGGGAGTAATCCCCTTGGCGGTTAAAACGCGGGGGACAGCGCGTACGTGCGTTTAAGCGGTGCTAGAGCTGTCTACGACCAATTGAGCGGCCTCGGCACCGGGATTCTCCAGG

#### Widom 601 –53I bottom strand sequence

ATGCACAGGATGTATATATCTGAC**I**CGTGCCTGGAGACTAGGGAGTAATCCCCTTGGCGGTTAAAACGCGGGGGACAGCGCGTACGTGCGTTTAAGCGGTGCTAGAGCTGTCTACGACCAATTGAGCGGCCTCGGCACCGGGATTCTCCAGG

#### Widom 601 –55I bottom strand sequence

ATGCACAGGATGTATATATCTG**I**CACGTGCCTGGAGACTAGGGAGTAATCCCCTTGGCGGTTAAAACGCGGGGGACAGCGCGTACGTGCGTTTAAGCGGTGCTAGAGCTGTCTACGACCAATTGAGCGGCCTCGGCACCGGGATTCTCCAGG

#### DNA preparation

The Widom 601 sequence^44^ was inserted into pET-51b vector, and the resulting plasmid was used as PCR template. The plasmid was transformed into Trans1-T1 *E. coli* cells and isolated using Plasmid Mini Kit I (Omega Bio-Tek). Damaged base-containing Widom 601 DNA was prepared by PCR with DI-containing primers. The primers used in this study were listed in Supplementary Table S2. A typical purification procedure required 10 mL of PCR reaction product. PCR reaction product was loaded onto a 5-mL Q beads 6FF FPLC column (Smart-Lifesciences, Changzhou) pre-equilibrated with 20 mM Tris-HCl (pH 8.0), and then eluted with linear gradient of NaCl (from 0 to 2 M, 20 mM Tris-HCl, pH 8.0). Peak fractions containing Widom 601 DNA were collected and concentrated using 10 K AmiconUltra-15 centrifugal filter unit (Merck).

Concentrated solution was supplemented with 1/10 volume of 3 M sodium acetate and 2 volumes of 100% ethanol to precipitate DNA. After 1 h of freezing at –40 °C, the mixture was centrifuged at 15000 rpm at 4 °C for 10 min, and the DNA pellet was washed with ice-cold 70% ethanol and dried in air. The resulting DNA pellet was stored at –80 °C for nucleosome reconstitution.

### Histone octamer preparation

Methods for expression and purification of *X. laevis* histones were adapted from previous studies^45,47^. In brief, H2A from *X. laevis* was cloned into pET-22b vector, which contained a 6× His tag and a TEV site at the N-terminus of H2A. H2B, H3 and H4 were cloned into pET-3a vectors.

Histone expression was induced with IPTG at 37 °C, and histone-expressing bacterial cells from 1 L H2A, 4 L H2B, 2 L H3 and 2 L H4 of bacterial cultures were mixed to achieve stoichiometry of 1:1:1:1. Mixed cells were lysed by sonication, and inclusion body was recovered by centrifugation and dissolved in 20 mM acetate, pH 5.2, 8 M guanidine hydrochloride, 10 mM DTT. Supernatants containing denatured histones were recovered by centrifugation, and transferred into dialysis bags for histone octamer refolding. Histone octamer was dialyzed against a buffer (20 mM Tris, pH 8.0, 2 M NaCl, and 2 mM β-mercaptoethanol) at 4 °C for three times, with each time more than 8 h. After dialysis, supernatant containing refolded histone octamer was centrifugated, loaded onto a 5-mL Ni-smart beads 6FF FPLC column (Smart-Lifesciences, Changzhou), and eluted with a linear gradient of imidazole mixed by (20 mM Tris-HCl, 2 M NaCl, 2 mM β-mercaptoethanol) and (20 mM Tris-HCl, 2 M NaCl, 2 mM β-mercaptoethanol, 500 mM imidazole). Peak fractions were collected and concentrated, and then loaded onto Superdex 200 10/300 GL (Cytiva) in buffer E (20 mM Tris-HCl, pH 7.5, 2 M NaCl, 1 mM EDTA, 1 mM DTT). Peak fractions were analyzed by SDS-PAGE, and fractions containing only histone octamer were pooled, concentrated and stored at –80 °C in small aliquots.

### Nucleosome assembly

Nucleosome assembly was performed as previously described^46,63^ with some modifications. Briefly, the caps of 1.5-mL Eppendorf tube were used to make dialysis button. Histone octamer and DI-containing Widom 601 DNA were mixed at a molar ratio of 1:1 in buffer E, and incubated for at least 0.5 h on ice. The mixture was then transferred into dialysis buttons sealed with dialysis membrane. Dialysis buttons were transferred into a dialysis bag filled with buffer E. The dialysis bag was dialyzed against buffer F (20 mM Tris-HCl, pH 7.5, 50 mM NaCl, 1 mM EDTA, 1 mM DTT) for 17 h at 4 °C. To complete NCP assembly, the dialysis buttons were dialyzed against fresh buffer F for additional 4 h. Assembled nucleosome was verified by 6% TBE-PAGE and used for AAG–NCP complex assembly.

### Preparation of AAG–NCP complex

All AAG–NCP complexes were prepared with the following method. Nucleosome was mixed with AAG protein at a molar ration of 1:50 in buffer C. The complex was stabilized by GraFix^64^ using TLS-55 rotor, the mixture was loaded onto a glycerol gradient of 20 mM HEPES, pH 7.5, 100 mM NaCl, 0.1 mM EDTA, 1 mM DTT, 20% glycerol (v/v) and 20 mM HEPES, pH 7.5, 100 mM NaCl, 0.1 mM EDTA, 1 mM DTT, 40% glycerol (v/v), 0.05% glutaraldehyde. The sample was centrifuged at 200,000× *g* for 17 h at 4 °C, and fractionated into 100-μL aliquots manually. 10 μL of 1 M Tris-HCl (pH 7.5) was added to each aliquot to quench cross-linking reaction. Each fraction was examined for AAG–NCP complex assembly by 6% TBE-PAGE. Fractions containing AAG–NCP complex were pooled and concentrated, and the glycerol removal and buffer exchange were achieved by ultrafiltration using 50 K AmiconUltra-0.5 centrifugal filter unit. A final buffer of 20 mM Tris-HCl, pH 7.5, 50 mM NaCl, 0.1 mM EDTA, 1 mM DTT was used for cryo-EM sample preparation.

### Cryo-EM sample preparation and data collection

AAG–NCP complexes concentrated to ~1.0 mg/mL were used for cryo-grid preparation. Quantifoil holey carbon Au R1.2/1.3 grids were glow-discharged, and 4 μL of the sample was applied to glow-discharged grids in Vitrobot Mark IV at 8 °C and 100% humidity. After waiting for 10 s, the grids were plunged into the liquid ethane for vitrification. Grids were screened on a Talos Arctica (ThermoFisher) 200 kV TEM equipped with a K2 detector (Gatan). Data collection was performed with an FEI Titan Krios G2 TEM operated at 300 kV with a Gatan K2 (GIF) direct electron detector. Automated data collection was done using SerialEM^65^. Data were collected with nominal magnification of 130,000× (corresponding to a calibrated pixel size of 1.052 Å), with defocus range of –1.2 μm to –1.7 μm. For each movie stack, a total of 32 frames were collected at a dose rate of 8 e^–^Å^–2^s^–1^ for 8 s.

### Image processing

For each dataset, the movie stacks were first subjected to beam-induced motion correction, electron-dose weighting and two-fold binning using MotionCor2^66^. Contrast transfer function (CTF) parameters of dose-weighted motion-corrected micrographs were estimated by program Gctf^67^. Around 400 particles were manually picked to generate an initial set of 2D template for automatic particle picking. Two rounds of reference-free 2D classification (25 iterations) were applied after particle auto-picking, and bad 2D classes were discarded. The initial 3D reference was produced using RELION3.1^68^. All subsequent processing steps were performed using RELION 3.1 unless otherwise stated. The image processing workflow was summarized in Supplementary Figs. S2–S5.

For the AAG–NCP^–30^ dataset, a total of 3814 raw micrographs were acquired, and 1,421,601 particles were auto-picked. After 2D classification, 1,249,347 particles were kept and subjected to two rounds of 3D classification with no symmetry imposed, which generated two major states. One is a free NCP (189,350 particles), called apo state. The other is the AAG-bounded state (126,881 particles), called post-catalytic state (confirmed in the model building). The particles from these two states were re-extracted and re-centered using a box size of 200 pixels for 3D refinement. Two individual soft-edged masks were used for high-resolution refinement, leading to two density maps at resolutions of 3.0 Å (apo state) and 3.1 Å (post-catalytic state) (gold-standard Fourier shell correlation 0.143 criteria). Application of CTF refinement and Bayesian polishing boosted the resolutions to 2.8 and 2.9 Å (Supplementary Fig. S2), respectively. To further improve the local density of the AAG region in the AAG-bounded state, another round of mask-based 3D refinement was performed. Final density maps were corrected for the modulation transfer function of the K2 Summit detector. The map sharpening was carried out by both RELION and DeepEMhancer^69^. The B factors used in RELION were automatically estimated through the post-processing procedure and the local resolution maps were generated using ResMap^70^ in RELION.

For the AAG–NCP^–50^ dataset, 1,583,682 particles were selected from 3918 raw images. After cleaning up the dataset by two rounds of 2D classification and one round of 3D classification, 460,662 particles were left for further processing. For the apo state, another round of 3D classification was applied and one class showing clear features was chosen and subjected to 3D refinement. Finally, the resolution of the apo state was pushed to 2.9 Å. For the post-catalytic state, all particles from good classes of the first round 3D classification were merged, and a small soft spherical mask centered at the extra density near SHL-5 of the nucleosome was generated to perform a focused 3D classification (skip alignment). A class (73,954 particles) exhibiting strong additional density was selected and further refined. The CTF refinement and Bayesian polishing procedures were subsequently applied to improve the resolution of the final map to 3.0 Å (Supplementary Fig. S3).

For the AAG–NCP^–53^ dataset, 4374 raw images were obtained and 1,711,509 particles were auto-picked. The data processing steps of AAG–NCP^–53AP^ were similar to those of the AAG–NCP^–50^ dataset. A set of 129,507 and 98,691 particles were used to generate the final density maps for the apo state and AAG-bound state at 2.8 Å and 3.1 Å, respectively (Supplementary Fig. S4).

For the AAG–NCP^–55^ dataset, 1,706,627 particles were auto-picked from 4363 raw micrographs. Similarly, the final map of the apo state was determined at 2.8 Å resolution, and the final map of the post-catalytic state was determined at 2.9 Å resolution (Supplementary Fig. S5).

### Model building and refinement

Cryo-EM structure of a canonical nucleosome (PDB: 7OHC)^50^ was used as the initial template for modeling. For the NCP^–30I^, the crystal structure was first docked into the density map using UCSF ChimeraX^71,72^, and structures of histones and DNA were adjusted manually using Coot^73^. The –30A was replaced with DI. For the AAG–NCP^–30AP^, the structures of the nucleosome (PDB: 7OHC) and AAG (PDB: 1BNK)^39^ were similarly modeled, and the –30A was replaced with AP site based on local density. These atomic models were refined in real space using Phenix^74^ with the geometry and secondary structure restraints applied. The refined structures were re-checked in Coot to adjust the side chain to proper locations. Final atomic models were evaluated using Molprobity^75^. The same modeling procedures were performed for other maps. Although the AAG regions in the AAG–NCP^–50AP^ and AAG–NCP^–53AP^ maps were fragmented in the final sharpened maps, the nucleosomal DNA and the inserted residue Tyr162 were clearly resolved. Therefore, the atomic model of AAG was docked into these two density maps by rigid body fitting. The statistics of data collection and model validation were summarized in Supplementary Table S3.

### Structural analysis

Three published NCP structures (EMD-12900, PDB: 7OHC^50^; EMD-11220, PDB: 6ZHX^51^; EMD-21970, PDB: 6WZ5^52^) were used as a reference. Initial structural analysis indicated a scaling factor between the published map and our maps. Therefore, we further calibrated the voxel sizes of the published map and our maps using the crystal structure of a canonical NCP (PDB: 1KX5)^76^ as a standard. Based on the calibration, the voxel size of the reference density map (EMD-12900) should be 1.06 Å, and the other two references were not modified (calibrated scaling factors less than 1%). Subsequently, the reference model (PDB: 7OHC) was adjusted by real-space refinement against the calibrated reference map using Phenix. The resulting refined model was used as the atomic model of the canonical NCP for structural analysis. Structural comparisons were performed using UCSF ChimeraX, and atom models were aligned by histone H3 using the matchmaker function. RMSD of nucleosomal DNA was calculated based on atoms from DNA backbone (C1′, C2′, C3′, C4′, C5′, O3′, O4′, O5′, P, OP1, OP2). Buried surface area between the nucleosomal DNA and the octameric histone core was determined using buried area function in UCSF ChimeraX.

## Supplementary information

Supplementary Information

Video1

Video2

Video3

video4

## Data Availability

Atomic coordinates of the associated structures have been deposited to the Protein Data Bank (PDB) with the following accession codes 7XFC (NCP^–30I^), 7XFH (AAG–NCP^–30AP^), 7XFI (NCP^–50I^), 7XFJ (AAG–NCP^–50AP^), 7XFL (NCP^–53I^), 7XFM (AAG–NCP^–53AP^), 7XFN (NCP^–55I^) and 7XNP (AAG–NCP^–55AP^), respectively. The corresponding maps have been deposited to the Electron Microscopy Data Bank (EMDB) under the following accession numbers EMD-33171 (NCP^–30I^), EMD-33172 (AAG–NCP^–30AP^), EMD-33173 (NCP^–50I^), EMD-33174 (AAG–NCP^–50AP^), EMD-33175 (NCP^–53I^), EMD-33176 (AAG–NCP^–53AP^), EMD-33177 (NCP^–55I^) and EMD-33322 (AAG–NCP^–55AP^), respectively. All data are available from the corresponding author.
