# Supplementary material for: Structural and mechanistic insights into the DNA glycosylase AAG-mediated base excision in nucleosome
Source: Cell Discov. 2023 Jun 20;9:62. doi: 10.1038/s41421-023-00560-0 (PMC10281986; doi:10.1038/s41421-023-00560-0)
Supplement: Supplementary file 1 — Supplementary Information [file 41421_2023_560_MOESM1_ESM.pdf]

Supplementary Information

for

Structural and mechanistic insights into the DNA glycosylase AAG-mediated base excision in nucleosome

Lvqin Zheng[1,2,3], Bin Tsai[1,3], Ning Gao[1,*]

[1]State Key Laboratory of Membrane Biology, Peking-Tsinghua Center for Life Sciences, School of Life Sciences, Peking University, Beijing 100871, China

[2]Academy for Advanced Interdisciplinary Studies, Peking University, Beijing 100871, China

[3]These authors contributed equally to this work.

[*]Correspondence to: gaon@pku.edu.cn

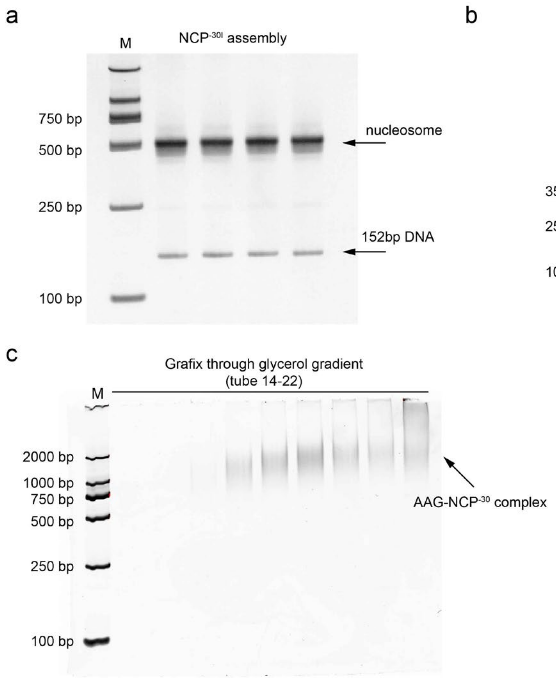

Supplementary Fig. S1 | Assembly of the AAG-NCP^{-30} complex.

a TBE-PAGE (6%) analysis of the reconstituted nucleosomes bearing a deoxyinosine at -30. The four lanes represent four independent assembly reactions. **b** SDS-PAGE analysis (4-20%) of the glycerol gradient fractions containing the AAG-NCP complexes. NCP, reconstituted NCP complexes; AAG-NCP, the reaction mixture. **c** TBE-PAGE analysis (6%) of the GraFix fractions containing the AAG-NCP^{-30} complexes.

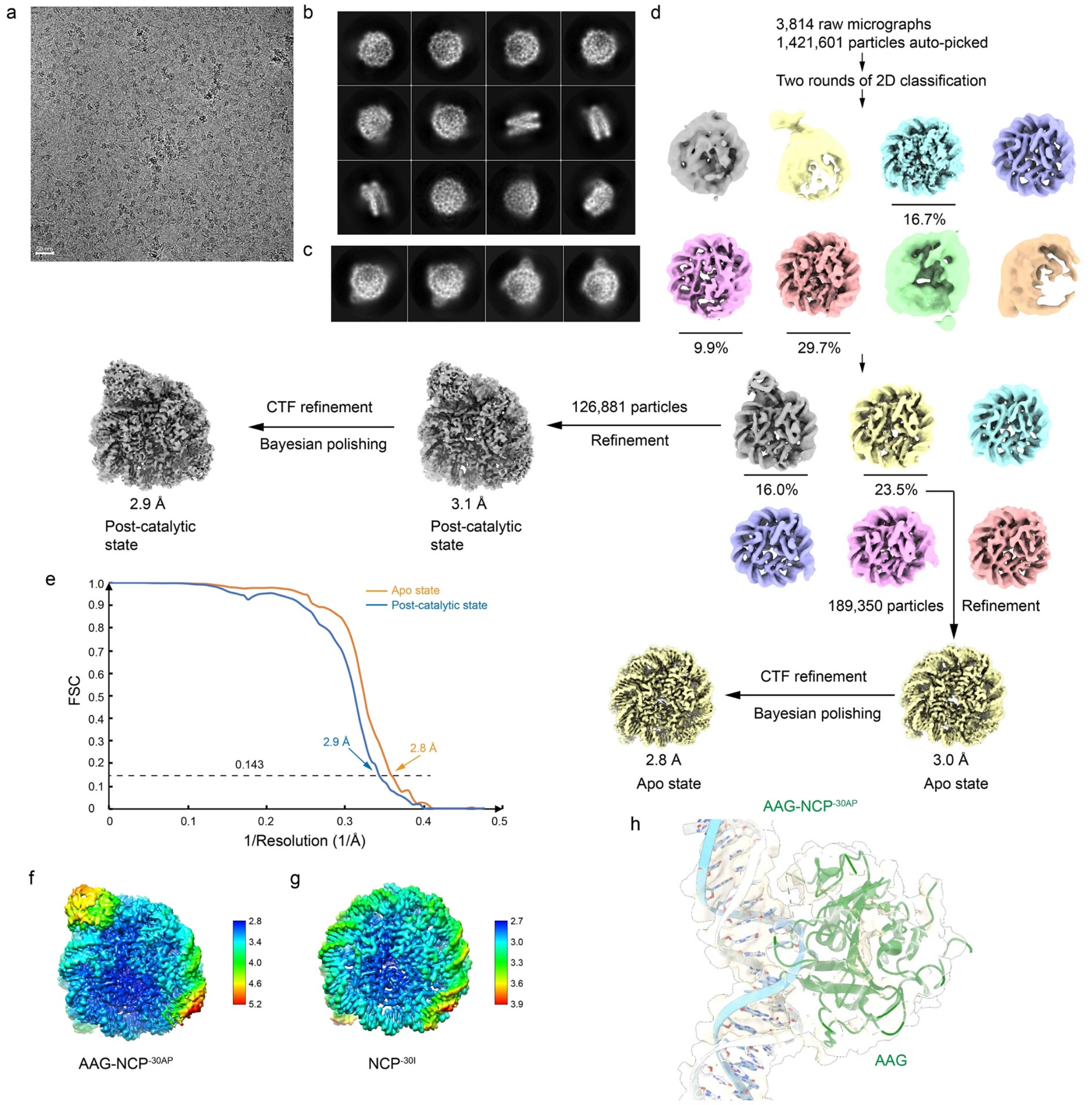

Supplementary Fig. S2 | Data processing of the AAG-NCP^{-30} dataset.

a A representative raw cryo-EM image of the AAG-NCP^{-30} sample. **b** Representative 2D classes of the AAG-NCP^{-30} dataset. **c** Representative 2D classes of the AAG-NCP^{-30AP} particles used for the final refinement of the AAG-NCP^{-30AP} complex. **d** Image-processing workflow of the AAG-NCP^{-30} dataset. **e** Gold-standard FSC curves of the final cryo-EM maps. **f** Final local resolution estimation of the AAG-NCP^{-30AP} map. **g** Final local resolution estimation of the NCP^{-30I} map. **h** Local cryo-EM density around the damaged base in the AAG-NCP^{-30AP} map.

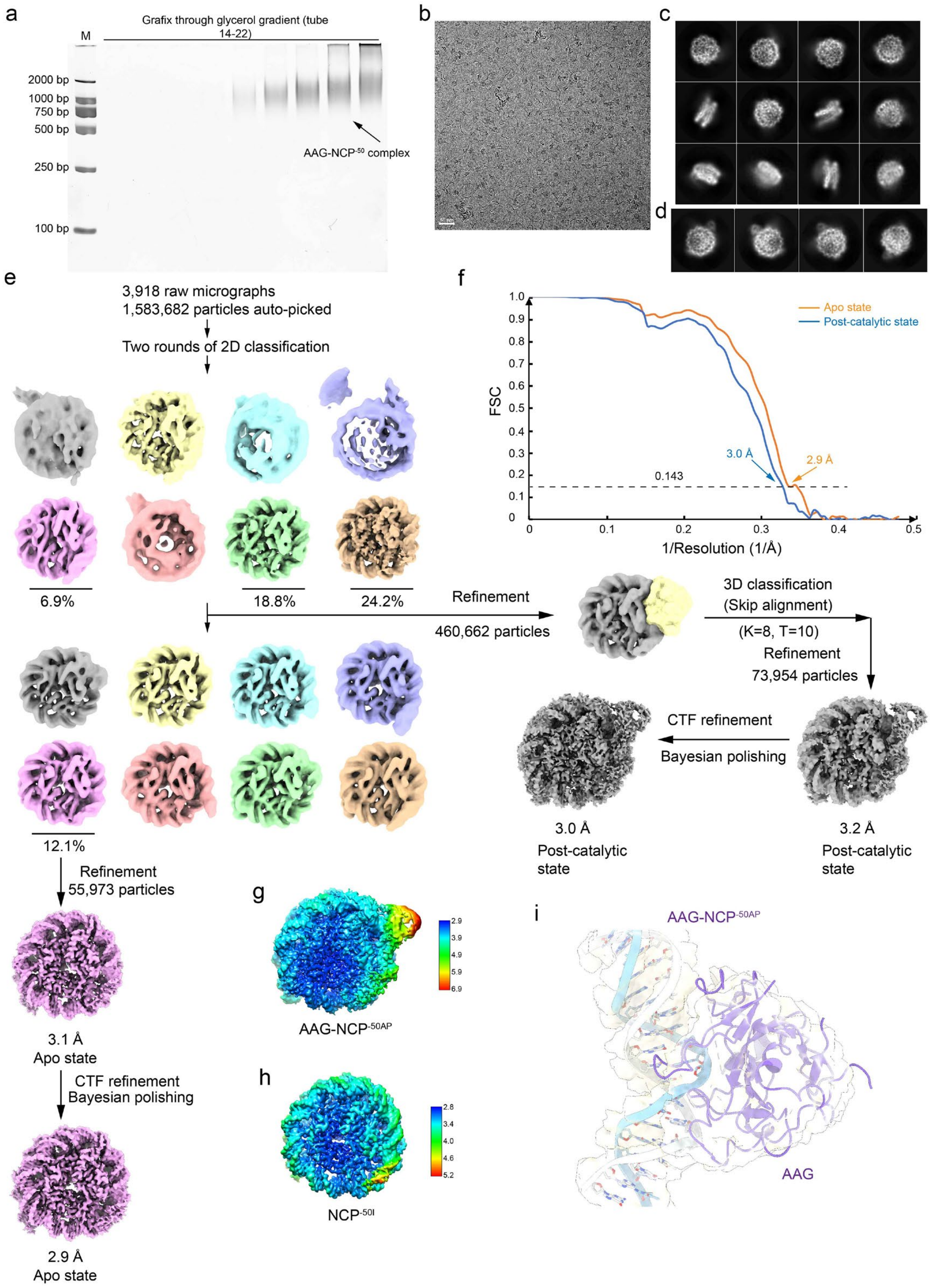

Supplementary Fig. S3 | Data processing of AAG-NCP^{-50} dataset.

a TBE-PAGE analysis (6%) of the GraFix fractions containing the AAG-NCP^{-50} complexes. **b** A representative raw cryo-EM image of the AAG-NCP^{-50} sample. **c** Representative 2D classes of the AAG-NCP^{-50} dataset. **d** Representative 2D classes of the AAG-NCP^{-50AP} particles used for the final refinement of the AAG-NCP^{-50AP}

complex. **e** Image-processing workflow of the AAG-NCP^{-50} dataset. **f** Gold-standard FSC curves of the final cryo-EM maps. **g** Final local resolution estimation of the AAG-NCP^{-50AP} map. **h,** Final local resolution estimation of the NCP^{-50I} map. **i** Local cryo-EM density around the damaged base in the AAG-NCP^{-50AP} map.

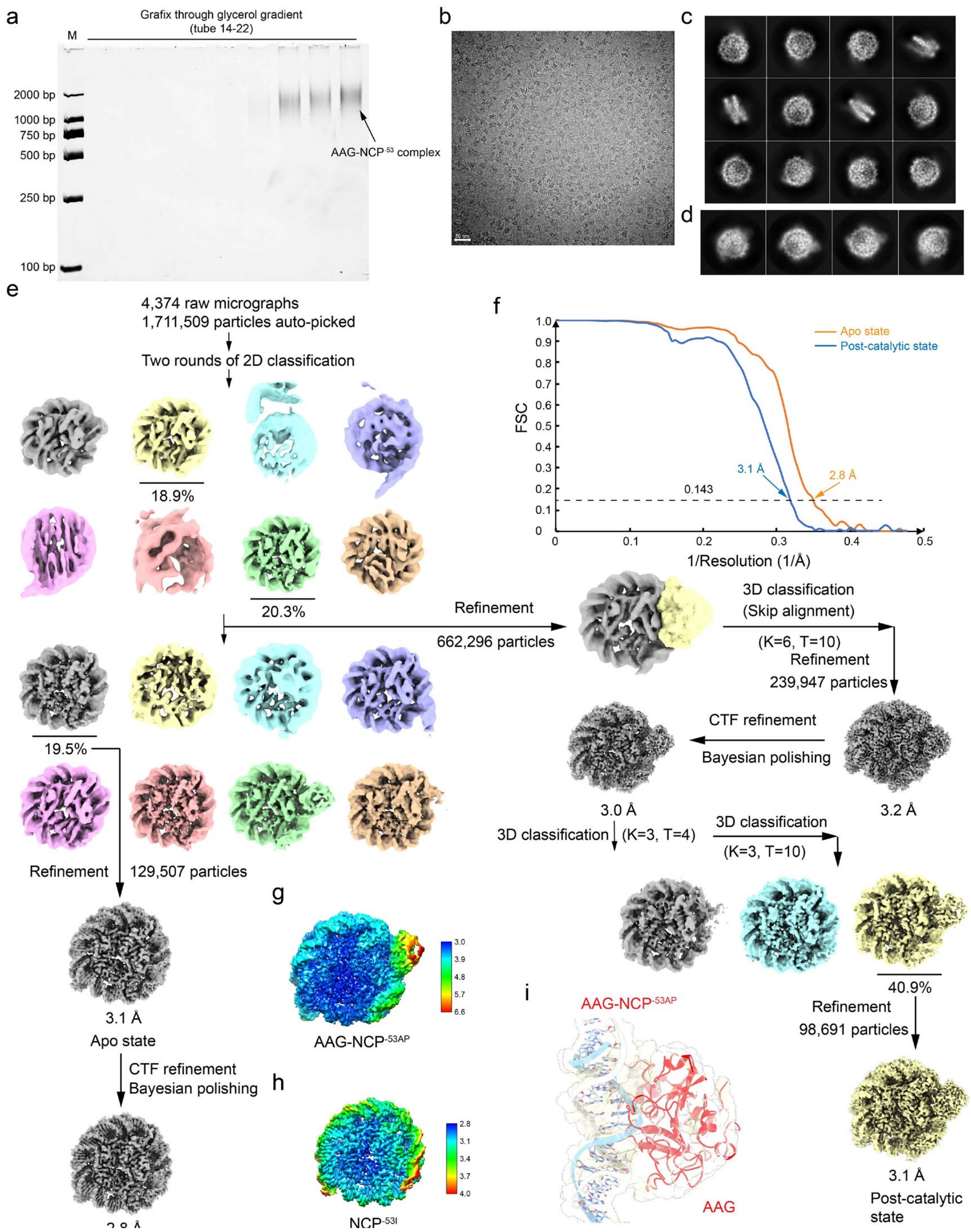

Supplementary Fig. S4 | Data processing of AAG-NCP^{-53} dataset.

a TBE-PAGE analysis (6%) of the GraFix fractions containing the AAG-NCP^{-53} complexes. **b** A representative raw cryo-EM image of the AAG-NCP^{-53} sample. **c** Representative 2D classes of the AAG-NCP^{-53} dataset. **d** Representative 2D classes of the AAG-NCP^{-53AP} particles used for the final refinement of the AAG-NCP^{-53AP} complex. **e** Image-processing workflow of the AAG-NCP^{-53} dataset. **f** Gold-standard FSC curves of the final cryo-EM maps. **g** Final local resolution estimation of the AAG-

NCP^{-53AP} map. **h** Final local resolution estimation of the NCP^{-53I} map. **i** Local cryo-EM density around the damaged base in the AAG-NCP^{-53AP} map.

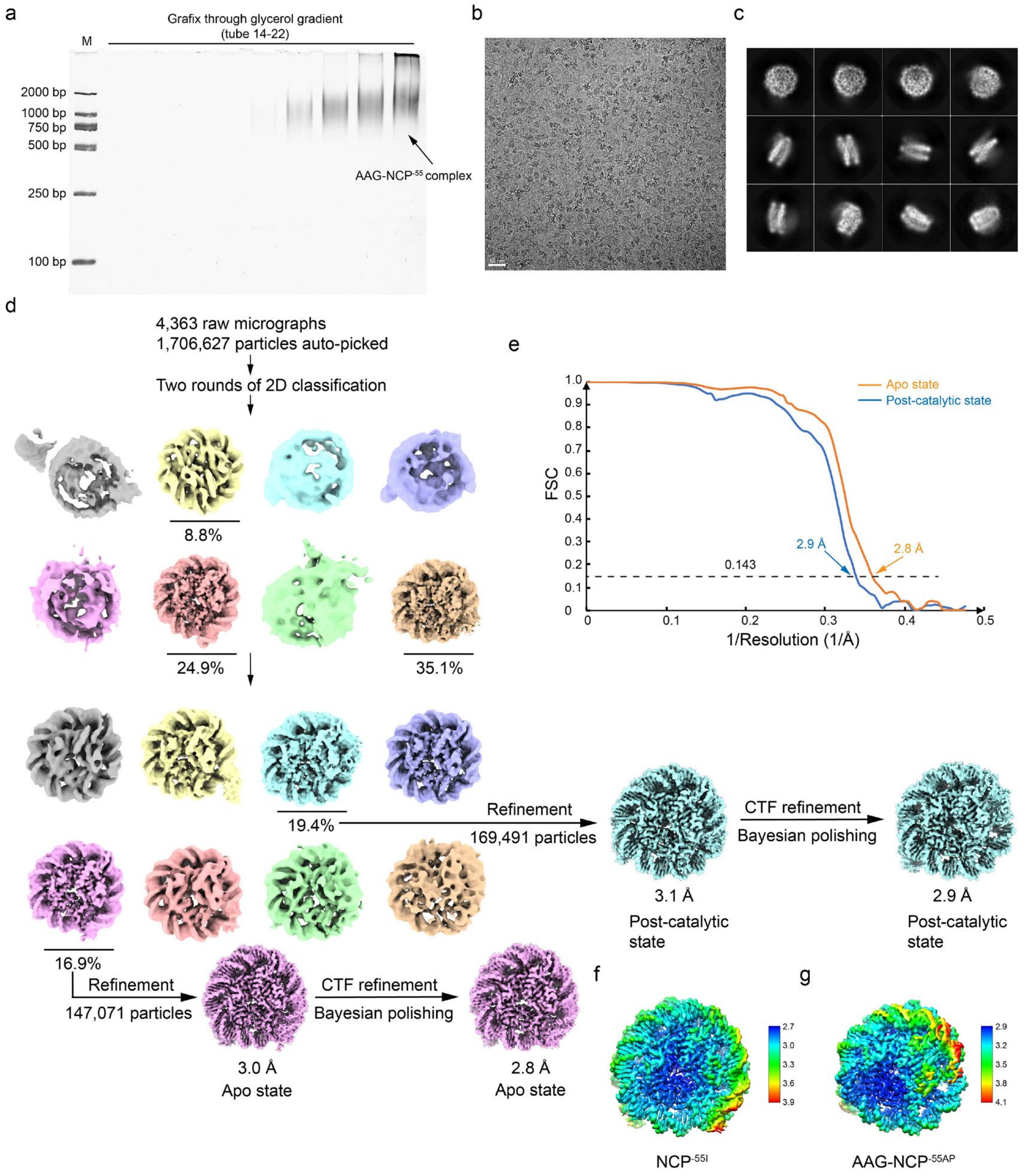

Supplementary Fig. S5 | Data processing of AAG-NCP^{-55} dataset.

a TBE-PAGE analysis (6%) of the GraFix fractions containing the AAG-NCP^{-55} complexes. **b** A representative raw cryo-EM image of the AAG-NCP^{-55} sample. **c** Representative 2D classes of the AAG-NCP^{-55} particles. **d** Image-processing workflow of the AAG-NCP^{-55} dataset. **e** Gold-standard FSC curve of the final cryo-EM maps. **f** Final local resolution estimation of the NCP^{-55I} map. **g** Final local resolution estimation of the AAG-NCP^{-55AP} map.

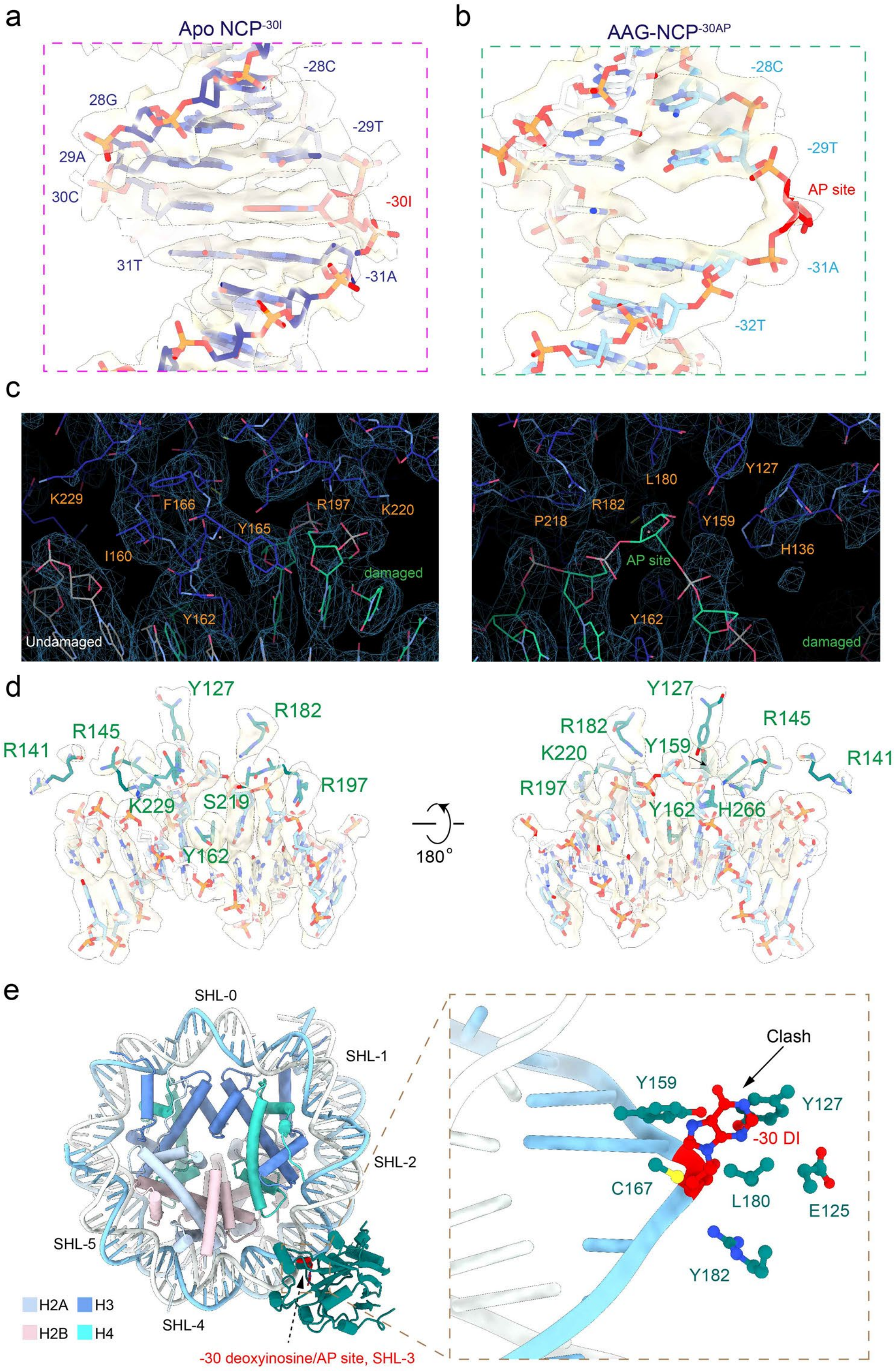

Supplementary Fig. S6 | Local density of the NCP^{-30I} and AAG-NCP^{-30AP} maps around the damaged base.

a Local cryo-EM density map of the nucleosomal DNA around deoxyinosine at -30 in the NCP^{-30I}. **b** Local cryo-EM density map of the nucleosomal DNA around the AP-site at -30 in the AAG-NCP^{-30AP}. **c-d** Local density of AAG around the damaged site in

the AAG-NCP^{-30AP} complex displayed in coot. **e** Superimposition of a deoxyinosine onto the model of the AAG-NCP^{-30AP}, highlighting the steric clash between the DI and AAG residues.

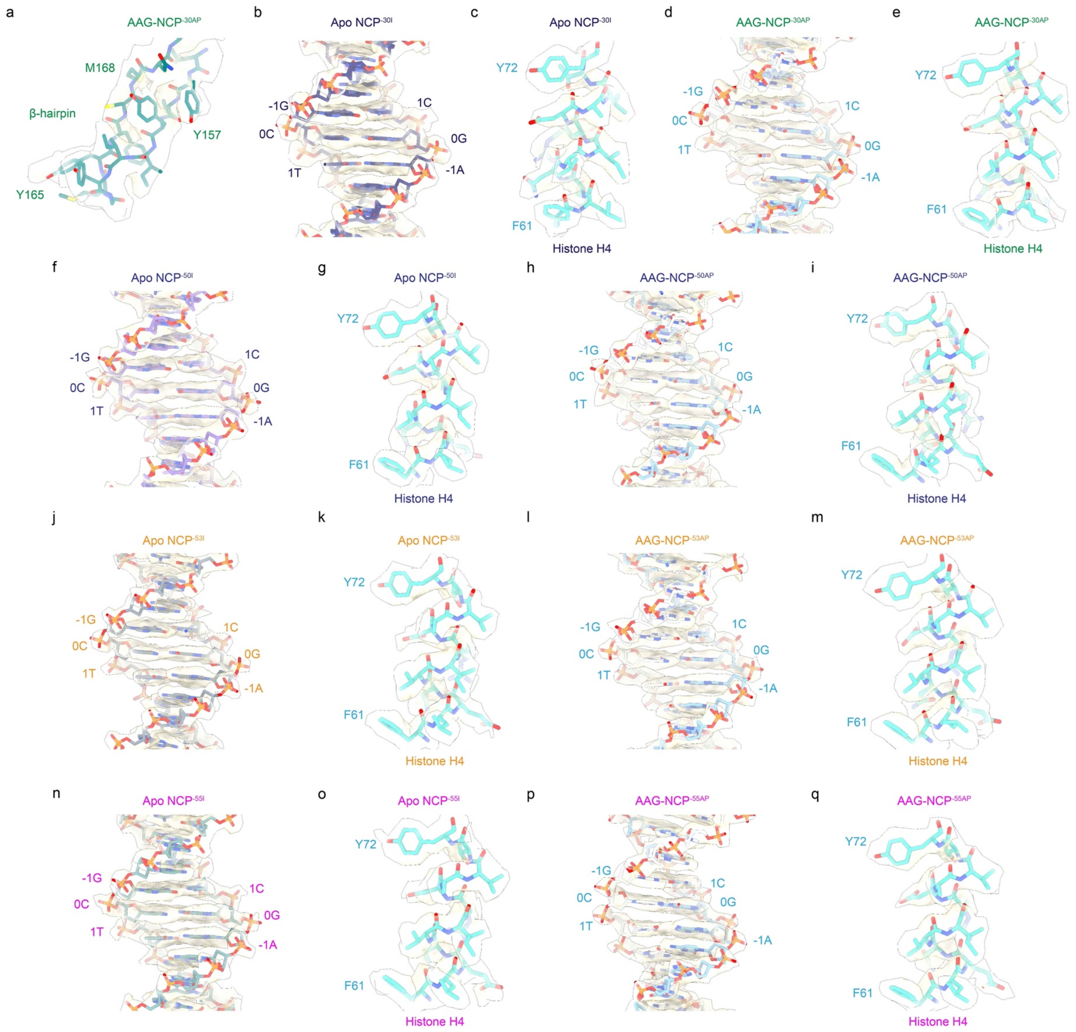

Supplementary Fig. S7 | Evaluation of cryo-EM density maps.

a-q Representative local cryo-EM density maps of AAG, nucleosomal DNA and Histone H4 for each cryo-EM map.

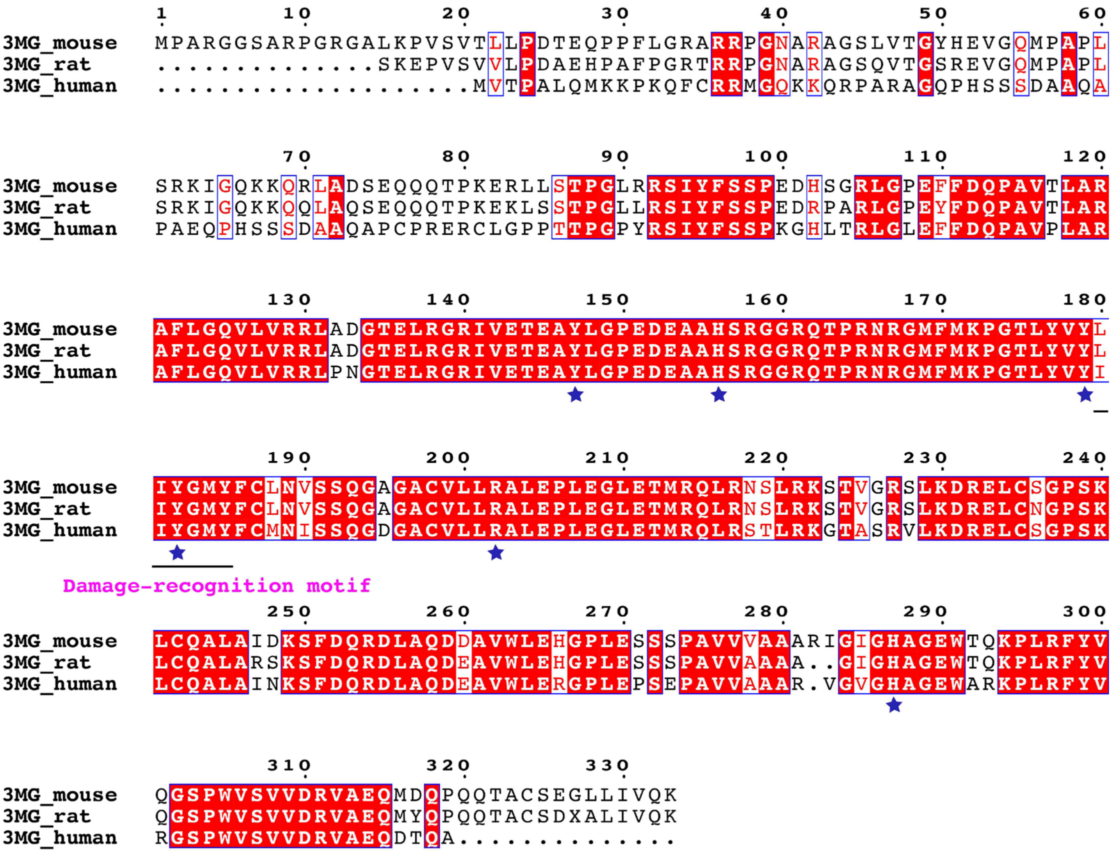

Supplementary Fig. S8 | Sequence alignment of DNA-3-methyladenine glycosylase from different species.

Alignment of DNA-3-methyladenine glycosylase sequence from *Home sapiens, Mus musculus* and *Rattus norvegicus*. The sequence is numbered according to the *Mus musculus one.* The damage-recognition motif is highlighted by a solid black line and the interacting residues of AAG are marked with blue pentagrams.

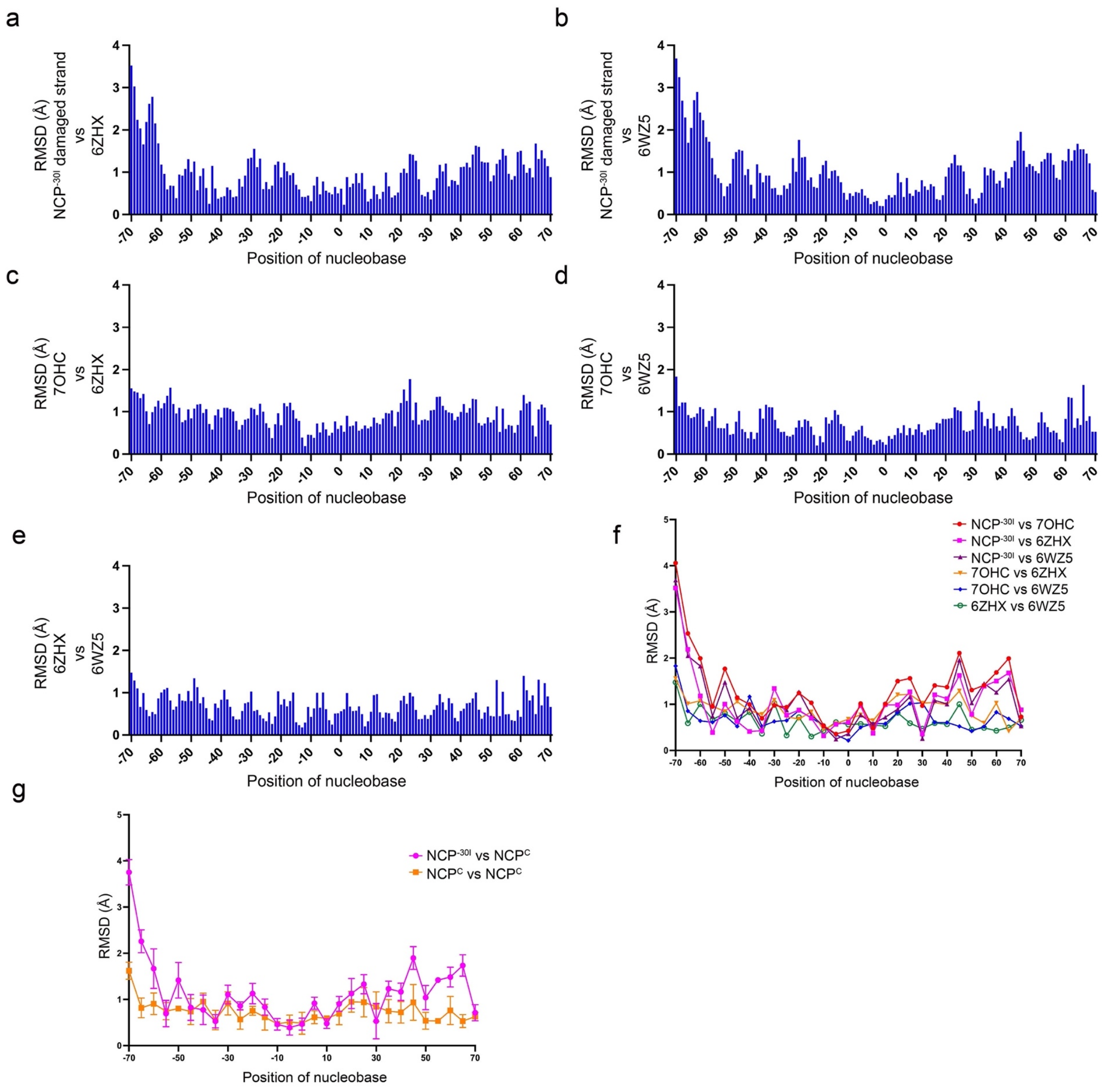

Supplementary Fig. S9 | RMSD plots for pairwise comparisons between the canonical and DI-containing nucleosomes (-30 position) and between different canonical nucleosomes.

a-b RMSDs of the DNA backbone between a canonical NCP (PDB: 6ZHX (**a**); 6WZ5 (**b**)) and the NCP^{-30I}. **c-e** RMSDs of DNA backbone between different canonical nucleosomes. 7OHC vs 6ZHX (**c**), 7OHC vs 6WZ5 (**d**), 6ZHX vs 6WZ5 (**e**). **f** Line chart of RMSD of DNA backbone between canonical NCPs (PDB: 7OHC; 6WZ5; 6ZHX) and the NCP^{-30I} or between different canonical nucleosomes. **g** Averaged RMSDs of DNA backbone between the NCP^{-30I} and each of the three representative canonical nucleosomes, or between different canonical nucleosomes as shown in (**f**).

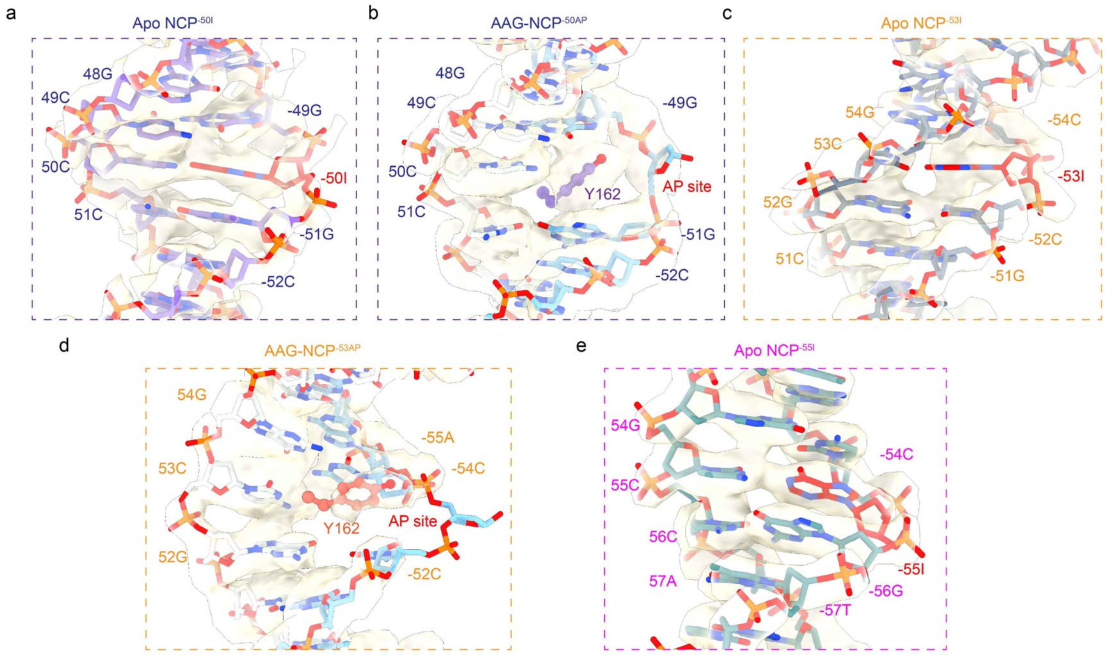

Supplementary Fig. S10 | Local density of the NCP^{-50I}, NCP^{-53I}, NCP^{-55I}, AAG-NCP^{-50AP}, and AAG-NCP^{-53AP} maps in the region of the damaged base.

a-b Local cryo-EM density map of the nucleosomal DNA around -50 in the NCP^{-50I} and AAG-NCP^{-50AP}. **c-d** Local cryo-EM density map of the nucleosomal DNA around -53 in the NCP^{-53I} and AAG-NCP^{-53AP}. **e** Local cryo-EM density map of the nucleosomal DNA around -55 in the NCP^{-55I}.

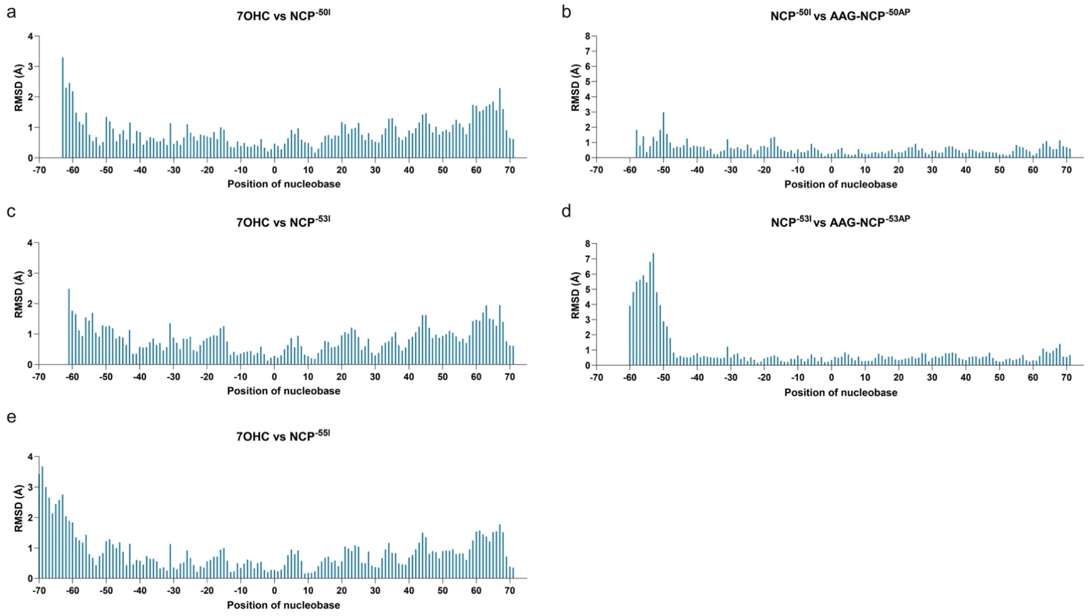

Supplementary Fig. S11| RMSD plots for pairwise comparisons of the nucleosomal DNA distortion.

a-b RMSD of the DNA backbone between a canonical NCP (PDB: 7OHC) and the NCP^{-50I}, and between the NCP^{-50I} and the AAG-NCP^{-50AP}. **c-d** RMSD of the DNA backbone between a canonical NCP (PDB: 7OHC) and the NCP^{-53I}, and between the NCP^{-53I} and the AAG-NCP^{-53AP}. **e** RMSD of the DNA backbone between a canonical NCP (PDB: 7OHC) and the NCP^{-55I}. The RMSD of each residue is plotted against residue number. Note that the terminal DNA (from ~60 to exit) of the NCP^{-50I}, AAG-NCP^{-50AP}, NCP^{-53I} and AAG-NCP^{-53AP} is relatively flexible in the maps and not modelled, and therefore not included for RMSD calculation.

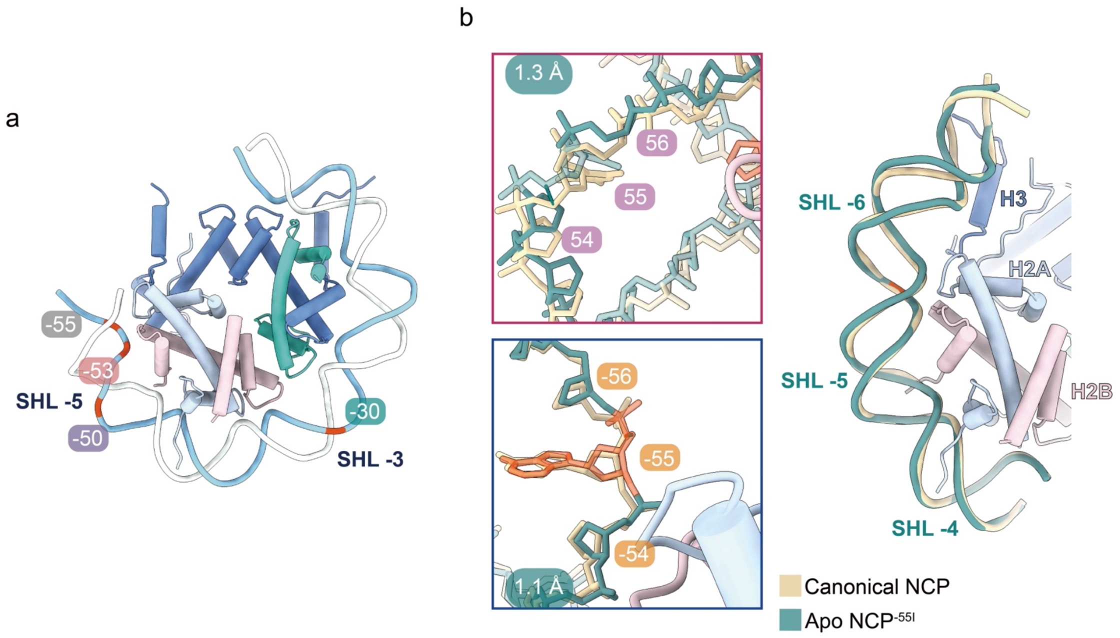

Supplementary Fig. S12 | Nucleosomal DNA deformation in the NCP^{-55I}.

a The position of -55 in the nucleosome. **b** The nucleosomal DNA perturbation of the NCP^{-55I} in comparison with a canonical NCP (PDB: 7OHC). RMSD of the DNA backbone is 1.3 Å for the damaged strand from 50 to 60, and 1.1 Å for the undamaged strand from -50 to -60.

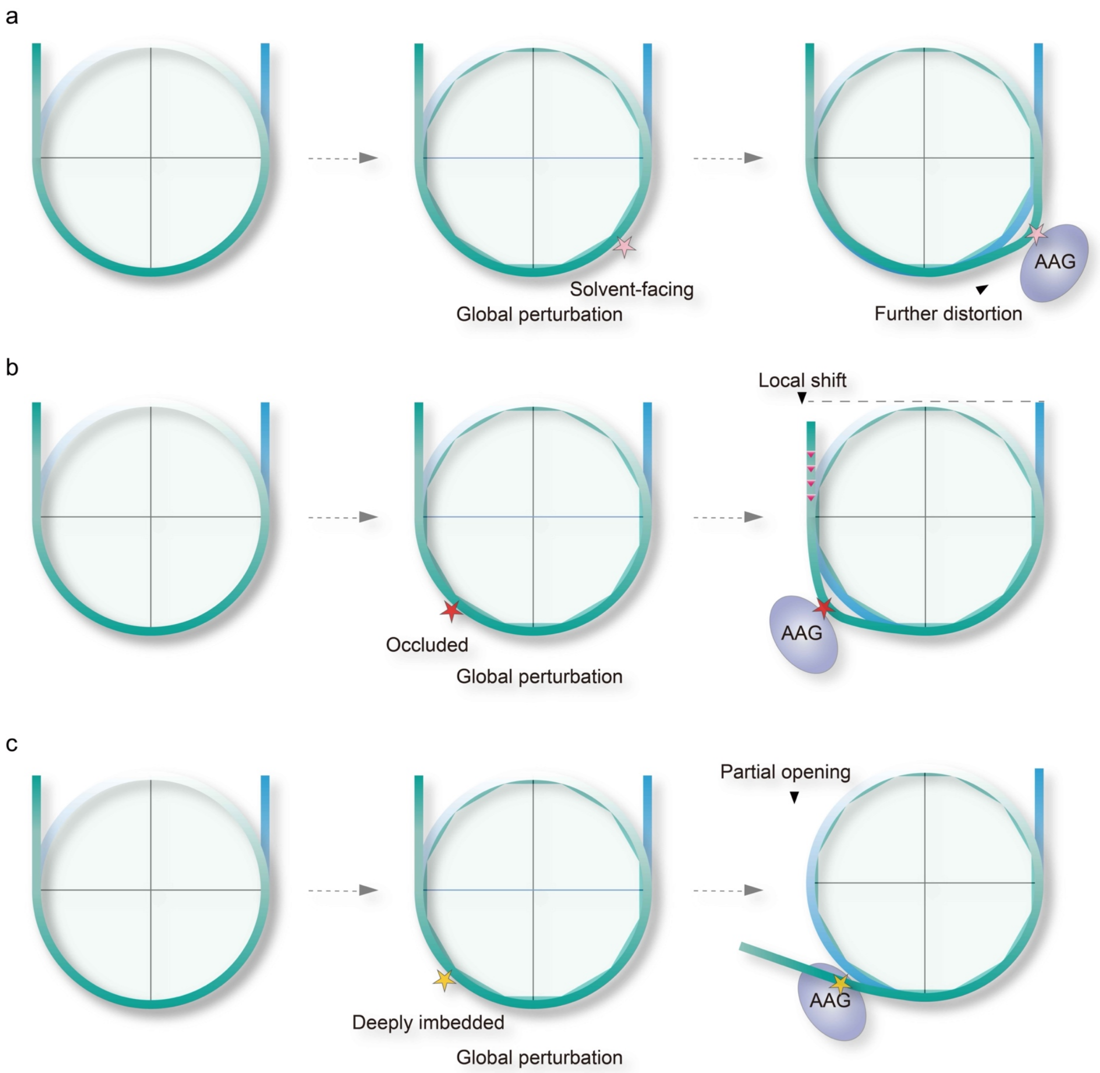

Supplementary Fig. S13| Models of AAG-mediated base excision in nucleosome.

a For solvent-facing position with high solution accessibility, by forming a stable AAG-NCP complex, AAG augments local DNA distortion to fulfill its function. **b** For occluded position with medium solution accessibility, the binding of AAG imposes drastic local DNA distortion and causes additional local register shift of DNA to avoid clash between flipped base and histones. **c** For deeply embedded position with low solution accessibility, local DNA distortion and translocation are insufficient to expose the damaged site, the global perturbation caused by deoxyinosine increases the possibility of spontaneous DNA unwrapping. AAG captures the damaged base in peeled-off DNA, and forms a stable AAG-NCP complex.

Supplementary Video S1. Local DNA distortion caused by AAG engagement in AAG-NCP^{-50AP}. Comparison between NCP^{-50I} and AAG-NCP^{-50AP}.

Supplementary Video S2. Close-up view of local DNA distortion caused by AAG engagement in AAG-NCP^{-50AP}. Close-up view of DNA distortion around -50 when comparing NCP^{-50I} to AAG-NCP^{-50AP}.

Supplementary Video S3. Local DNA distortion caused by AAG engagement in AAG-NCP^{-53AP}. Comparison between NCP^{-53I} and AAG-NCP^{-53AP}.

Supplementary Video S4. Close-up view of local DNA distortion caused by AAG engagement in AAG-NCP^{-53AP}. Close-up view of DNA distortion around -53 when comparing NCP^{-53I} to AAG-NCP^{-53AP}.

Supplementary Table S1. Buried surface area between nucleosomal DNA and Octameric histone core.

Buried Surface Area (Å^2) (DNA vs Histone core)	-30 dataset (DNA: -59 to 72)	-50 dataset (DNA: -58 to 72)	-53 dataset (DNA: -60 to 72)	-55 dataset (DNA: -70 to 72)
Canonical NCP (PDB: 7OHC),	7141.4	7133.6	7141.4	7516.9
Apo NCP	6195.4	6247.8	6075.4	6744.8
AAG-NCP complex	6165.8	5976.5	5897.2	N.A.

Supplementary Table S2. Sequences of the oligonucleotides used as primers for PCR amplification

601-top F	CCTGGAGAATCCCGGTGCCGA
-30I R	ATGCACAGGATGTATATATCTGACACGTGCCTGGAGA CTAGGGAGTA**I**TCCCCTTGGC
-50I R	ATGCACAGGATGTATATATCTGACACG**I**GCCTGGAGA CTA
-53I R	ATGCACAGGATGTATATATCTGAC**I**CGTGCCTGGA
-55I R	ATGCACAGGATGTATATATCTG**I**CACGTGCCTGGA

Supplementary Table S3. Cryo-EM data collection, refinement and validation statistics

	Apo NCP^{-30I} (EMDB-33171) (PDB 7XFC)	AAG-NCP^{-30AP} (EMDB-33172) (PDB 7XFH)	Apo-NCP^{-50I} (EMDB-33173) (PDB 7XFI)	AAG-NCP^{-50AP} (EMDB-33174) (PDB 7XFJ)
**Data collection and processing**				
Magnification	130,000	130,000	130,000	130,000
Voltage (kV)	300	300	300	300
Electron exposure (e–/Å^2)	64	64	64	64
Defocus range (μm)	-1.2 to -1.7	-1.2 to -1.7	-1.2 to -1.7	-1.2 to -1.7
Pixel size (Å)	1.052	1.052	1.052	1.052
Symmetry imposed	C1	C1	C1	C1
Initial particle images (no.)	1,421,601	1,421,601	1,583,682	1,583,682
Final particle images (no.)	189,350	126,881	55,973	73,954
Map resolution (Å)	2.8	2.9	2.9	3.0
FSC threshold	0.143	0.143	0.143	0.143
Map resolution range (Å)	2.7-3.9	2.8-5.2	2.8-5.2	2.9-6.9
**Refinement**				
Initial model used (PDB code)	7OHC	7OHC, 1BNK	7OHC	7OHC,1BNK
Model resolution (Å)	2.8	2.9	2.9	3.0
FSC threshold	0.143	0.143	0.143	0.143
FSC map vs model (0.5)	2.8	3.0	3.0	3.1
Map sharpening *B* factor (Å^2)	-65.644	-64.544	-51.629	-70.71
Model composition				
Non-hydrogen atoms	11,802	12,971	11526	12807
Protein residues	750	957	751	943
Ligands	1	1	1	1
B factors (Å^2)				
Protein	34.90	61.07	41.06	73.44
Ligand	58.43	113.51	80.71	145.12
R. m. s. deviations				
Bond lengths (Å)	0.005	0.005	0.005	0.006
Bond angles (°)	0.798	0.764	0.800	0.778
Validation				
MolProbity score	1.01	1.16	1.03	1.22
Clash score	2.30	2.35	2.49	2.38
Poor rotamers (%)	0.00	0.00	0.00	0.00
Ramachandran plot				
Favored (%)	98.64	97.22	98.37	96.74
Allowed (%)	1.36	2.78	1.63	3.26
Disallowed (%)	0.00	0.00	0.00	0.00

	Apo NCP-53I (EMDB-33175) (PDB 7XFL)	AAG-NCP-53AP (EMDB-33176) (PDB 7XFM)	Apo NCP-55I (EMDB-33177) (PDB 7XFN)	AAG-NCP-55AP (EMDB-33322) (PDB 7XNP)
**Data collection and processing**				
Magnification	130,000	130,000	130,000	130,000
Voltage (kV)	300	300	300	300
Electron exposure (e–/Å^2)	64	64	64	64
Defocus range (μm)	-1.2 to -1.7	-1.2 to -1.7	-1.2 to -1.7	-1.2 to -1.7
Pixel size (Å)	1.052	1.052	1.052	1.052
Symmetry imposed	C1	C1	C1	C1
Initial particle images (no.)	1,711,509	1,711,509	1,706,627	1,706,627
Final particle images (no.)	129,507	98,691	147,071	169,491
Map resolution (Å)	2.8	3.1	2.8	2.9
FSC threshold	0.143	0.143	0.143	0.143
Map resolution range (Å)	2.8-4.0	3.0-6.6	2.7-3.9	2.9-4.1
**Refinement**				
Initial model used (PDB code)	7OHC	7OHC, 1BNK	7OHC	7OHC
Model resolution (Å)	2.8	3.1	2.8	2.9
FSC threshold	0.143	0.143	0.143	0.143
FSC map vs model (0.5)	3.0	3.1	2.8	3.0
Map sharpening B factor (Å^2)	-65.573	-110.266	-74.704	-67.841
Model composition				
Non-hydrogen atoms	11,428	12,916	11,833	10,945
Protein residues	749	946	753	746
Ligands	1	1	1	1
B factors (Å^2)				
Protein	38.03	83.89	33.42	10.08
Ligand	95.58	196.11	60.50	
R.m.s. deviations				
Bond lengths (Å)	0.005	0.004	0.005	0.005
Bond angles (°)	0.772	0.958	0.791	0.748
Validation				
MolProbity score	0.95	1.18	0.97	1.03
Clashscore	1.89	3.08	2.02	2.36
Poor rotamers (%)	0.00	0.00	0.00	0.00
Ramachandran plot				
Favored (%)	98.36	97.62	98.24	97.95
Allowed (%)	1.64	2.38	1.76	2.05
Disallowed (%)	0.00	0.00	0.00	0.00
